# Identification of novel acetylcholinesterase inhibitors designed by pharmacophore-based virtual screening, molecular docking and bioassay

**DOI:** 10.1038/s41598-018-33354-6

**Published:** 2018-10-08

**Authors:** Cheongyun Jang, Dharmendra K. Yadav, Lalita Subedi, Ramu Venkatesan, Arramshetti Venkanna, Sualiha Afzal, Eunhee Lee, Jaewook Yoo, Eunhee Ji, Sun Yeou Kim, Mi-hyun Kim

**Affiliations:** Gachon Institute of Pharmaceutical Science and Department of Pharmacy, College of Pharmacy, Gachon University, Yeonsu-gu, Incheon Republic of Korea

## Abstract

In this study, pharmacophore based 3D QSAR models for human acetylcholinesterase (AChE) inhibitors were generated, with good significance, statistical values (r^2^_training_ = 0.73) and predictability (q^2^_training_ = 0.67). It was further validated by three methods (Fischer’s test, decoy set and Güner-Henry scoring method) to show that the models can be used to predict the biological activities of compounds without costly and time-consuming synthesis. The criteria for virtual screening were also validated by testing the selective AChE inhibitors. Virtual screening experiments and subsequent *in vitro* evaluation of promising hits revealed a novel and selective AChE inhibitor. Thus, the findings reported herein may provide a new strategy for the discovery of selective AChE inhibitors. The IC_50_ value of compounds **5c** and **6a** presented selective inhibition of AChE without inhibiting butyrylcholinesterase (BChE) at uM level. Molecular docking studies were performed to explain the potent AChE inhibition of the target compounds studies to explain high affinity.

## Introduction

Alzheimer’s disease (AD) is a representative degenerative brain disease that is characterized by clinical signs such as declining cognitive functional, thinking skills and understandings. The pathogenesis and etiology of this ailment remain unclear. Typically, the extracellular aggregation of amyloid plaques has been considered as an indicator of AD^1^. AD was first documented more than a century ago, but research into its root causes, symptoms, risk factors and treatment has achieved momentum most effective inside the beyond few decades. Despite the fact that research has discovered some of biological goals targets against AD which includes acetylcholinesterase (AChE), N-methyl-d-aspartate (NMDA) receptor, glycogen synthase kinase 3 (GSK3), cyclin-dependent kinase 5 (CDK5), secretase, etc. but the specific drug molecules against these targets showing a complete cure of the disease stay unknown^1,2^. AD can accompany a decline in the level of the neurotransmitter acetylcholine (ACh) and thus to raise the level of ACh, a key enzyme in the breakdown of the ACh i.e. AChE can be targeted^3^. Acetylcholinesterase, (AChE; E.C. 3.1.1.7) which is among the most efficient enzymes with a turnover number variety of >10^4^ s^−1^, is one of the potential targets, which has led to some palliative drugs approved for the treatment of AD^4,5^. The most prominent and known neuropathological characteristics found in AD patients are the presence of amyloid beta (Aβ) plaques and neurofibrillary tangles within the brain^6^. It is found that AChE present in the cholinergic terminals accelerates this Aβ aggregation^7^. More recent studies suggest that the AChE-Aβ complex boost the Aβ dependent deregulation of intracellular Ca^2+^ plus mitochondrial disordered in hippocampal neurons, which causes more deterioration than Aβ alone^8^. The FDA approved only four acetyl cholinesterase inhibitors (AChEIs) for the treatment of this disease and they are classified into two therapeutic classes: N-methyl-D-aspartic acid (NMDA) antagonists (meantime) and acetylcholinesterase inhibitors (donepezil, galantamine and rivastigmine). Other trials to find new drug targets are ongoing, along with trials investigating the use of anti-amyloid immunotherapy and nerve growth factor (NGF) gene therapy^9^. However, new therapeutic targets need to be determined to develop new drugs to control symptoms and to modify disease.

In addition to recent studies, the β-adrenergic receptor (β-AR) has been highlighted as a new therapeutic target for Alzheimer’s disease. The β-AR may be stimulated with the aid of stress^10^. Activated β-AR enhances γ-secretase activity and stimulates the accretion of amyloid beta protein (Aβ) within the interstices of the brains^11,12^. It also increases cAMP levels, enhances amyloid precursor protein levels in astrocytes^13^ and promotes amyloid beta protein production. Furthermore, a recent research has reported central nervous system (CNS) side effects related to β-AR blockers such as sleep disorders, fatigue, nightmares and hallucinations^14^. The lipophilicity and permeability of the blood brain barrier(BBB) had been taken consideration as potential factors for these CNS side effects^15^. They can easily penetrate BBB and bind with non-adrenergic receptor in the CNS and disturb the serotonin pathway^16^. Also, it can decrease melatonin secretion and cause nightmare^17^. Other side effects have also been reported; however, the mechanisms underlying these effects have not been determined^18–21^. In recent years, many reports have proven that β-adrenoceptor antagonists also have neuroprotective effects^22–25^. Many researchers have tried to understand protective effects of β-adrenoceptor antagonists against hypoxia and perfusion after traumatic brain injury. However, the mechanisms underlying this effect are unknown. For decades, many studies have reported that increased epinephrine and norepinephrine levels protected neurons^26–28^. If β-blockers could simultaneously inhibit AChE, they could be used to treat AD and manipulate signs and symptoms.

For *in silico* rational design of new scaffolds, we have conducted ‘de novo design/core-hopping’^29^, ‘side-chain hopping’^30^, in addition to prediction of binding mode through MD simulations^31^ in structure-based prediction models. Similarly, like our previous shape-based QSAR model^32^, we could consider developing ligand-based predictive models to extract information regarding distinct structural features required for ligand-receptor interaction^33^. The database can be initially screened for drug-like molecules by applying different rational filters such as the Lipinski’s Rule of five^34–36^ and drug-like adsorption, distribution, metabolism, excretion and toxicity (ADMET) properties^36–38^. Subsequently, it could be further subjected to molecular docking interaction based screening. Docking technique commonly utilizes an energy-based scoring function to acquire the most favorable ligand orientation and conformation, required for binding within the active site^39–42^.

**Figure 1:**
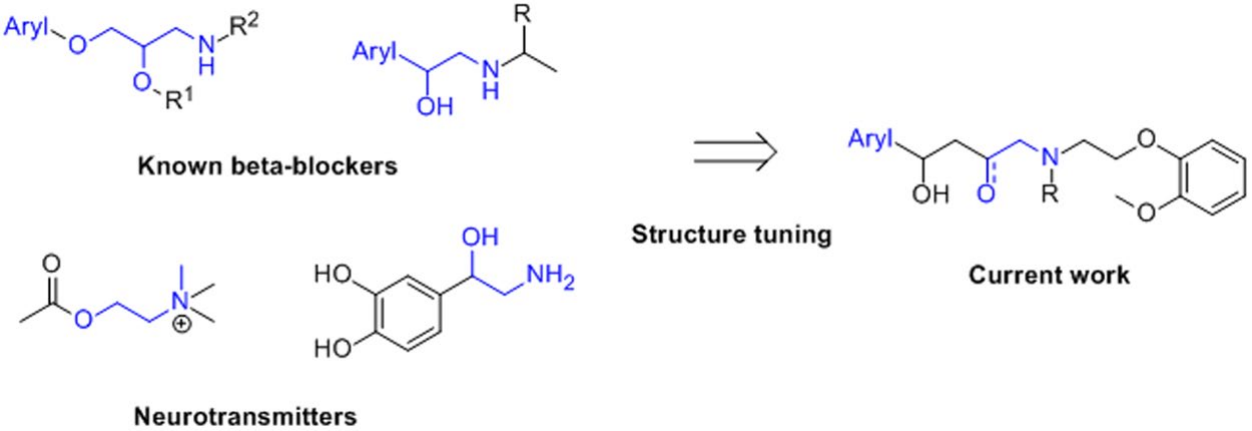
Strategy and structures of beta-blockers and neurotransmitters.

In this study, we developed pharmacophore models based on AChE inhibitors accrued from ChEMBL database^43^. The pharmacophore features were used to identify potent AChE inhibitors in addition to make clear the quantitative structure activity relationship for known AChE inhibitors. The best quantitative model was used as 3D search queries for screening the ChEMBL databases to identify new inhibitors of AChE that may block both the catalytic and peripheral anionic sites. Once identified, the hit compounds were subsequently subjected to filtering through molecular docking to refine the retrieved hits. The virtual screening approach, in the combination with pharmacophore modeling, molecular docking and consensus scoring function can be used to identify and design novel AChE inhibitors with higher selectivity. Based on pharmacophore modeling we have designed and synthesized a series of six benzyl-2-(2-methoxyphenoxy)-ethyl amine β-blocker and neurotransmitter following this protocol shown in Figure 1. β-AR antagonists share a common structure; a β-ethanolamine linked with an aromatic ring, which serves as a critical scaffold for its function as a β-AR antagonist^44,45^. The side chain with the aromatic ring is variable. Thus, the aim of this work was to investigate the interactions among the potential inhibitors and AChE by pharmacophore modeling, virtual screening, molecular docking simulation and *in vitro* bio-assays techniques in order to contribute to the elucidation of its mechanisms action.

## Results and Discussion

### Pharmacophore and 3D-QSAR models

A 3D-QSAR model for the set of 82 compounds was developed using PHASE module of Schrodinger. A common pharmacophore hypothesis was built with binding sites to generate six variants among given data, which generated ten hypotheses. The scoring method become applied to hypotheses obtained based on different grounds such as actives, inactive, volume and selectivity. Best hypothesis was based on best post-hoc survival score combining active and inactive survival scores. The different scoring parameters for best hypothesis AAHPRR.15 are given in Table 1.

**Table 1:** Statistic variations of the pharmacophore model.

ID	Survival	Site	Vector	Volume	Selectivity	# Matches
AAHPRR.10	3.529	0.76	0.993	0.778	3.03	7
AAHPRR.9	3.529	0.76	0.993	0.778	3.03	7
AAHPRR.11	3.352	0.61	0.986	0.76	3.019	8
AAHPRR.15	3.294	0.65	0.991	0.657	3.026	7
AAHPRR.7	3.272	0.61	0.987	0.672	3.034	7
AAHPRR.8	3.272	0.61	0.987	0.672	3.034	7
AAHPRR.26	3.089	0.48	0.923	0.689	3.203	7
AAHPRR.38	3.08	0.53	0.94	0.613	3.24	6
AAHPRR.24	3.08	0.53	0.94	0.613	3.24	6
AAHPRR.16	3.058	0.38	0.979	0.702	3.044	6

**Figure 2:**
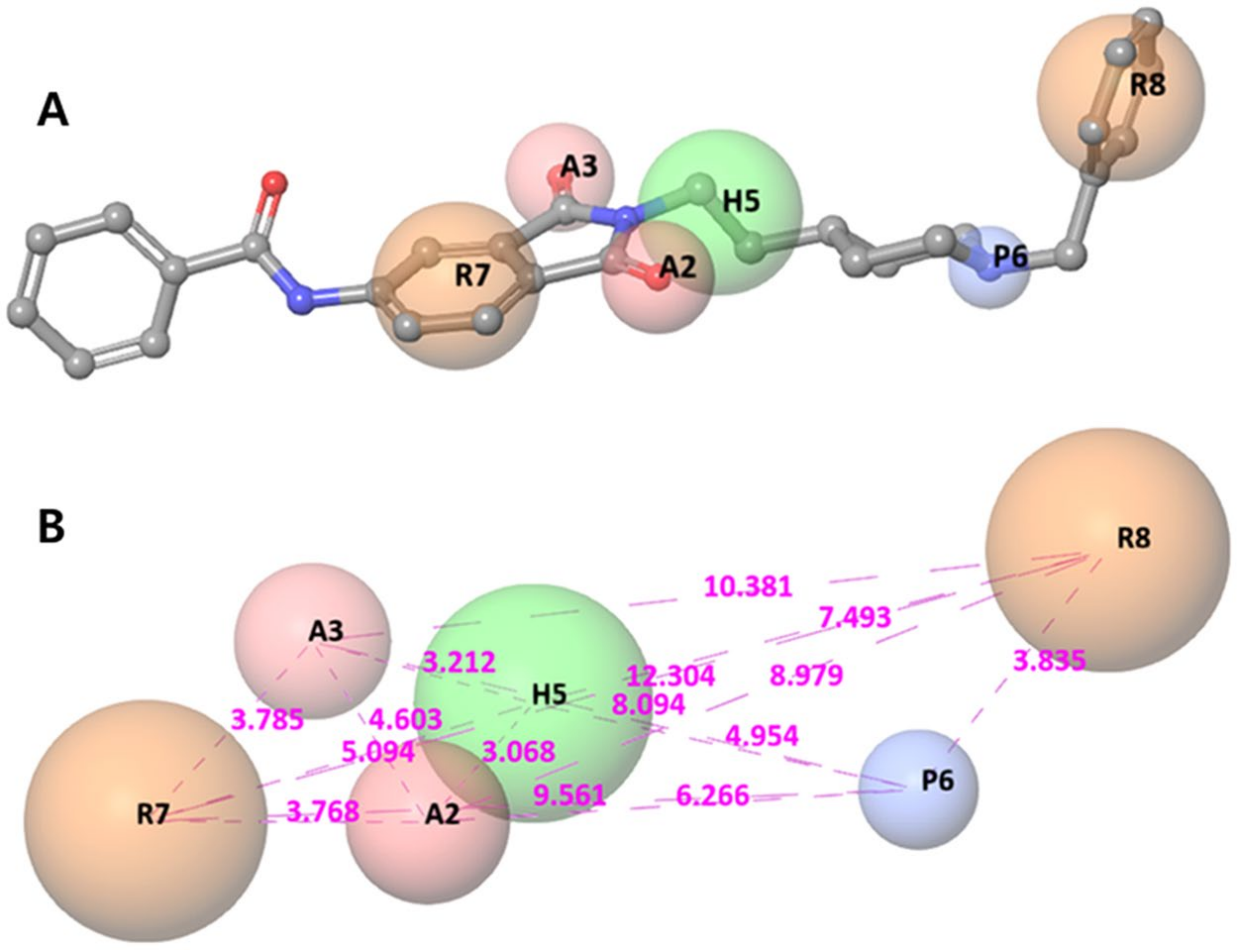
Best pharmacophore model AAHPRR.15 of AChE inhibitor with a reference ligand and its distance. Pharmacophore features are coded with different colors: 2 hydrogen bond acceptors (A2, A3; pink), 1 positive regions (P6; blue), 1 hydrophobic regions (H5; green) and 2 aromatic ring (R7, R8; orange).

Based on sites, maximum of six features were allowed to develop hypotheses and 10 common hypotheses were reported in all 82 compounds. The best fitted Model AAHPRR.15 (R^2^ = 0.73, Q^2^ = 0.67 and F = 149) consists of two hydrogen bond acceptor, one hydrophobic two aromatic ring features and one polar point (Fig. 2A,B) with the survival score (3.294). It’s also evident from comparison that survival minus inactive score, which deducts the inactive features from the hypothesis, was also decisively highest for the hypothesis AAHPRR.15. The principal attribute and the factor of difference between active and inactive is due to the interstitial site distances as evident by the pharmacophore hypothesis AAHPRR.15 alignments over active compounds (pIC_50_ > 6.9) and inactive compounds (pIC_50_ < 6.9) in Fig. 2A,B, respectively. Among pharmacophore features, intersite distances and angles shown in Supplementary Table 1 between site points.

**Figure 3:**
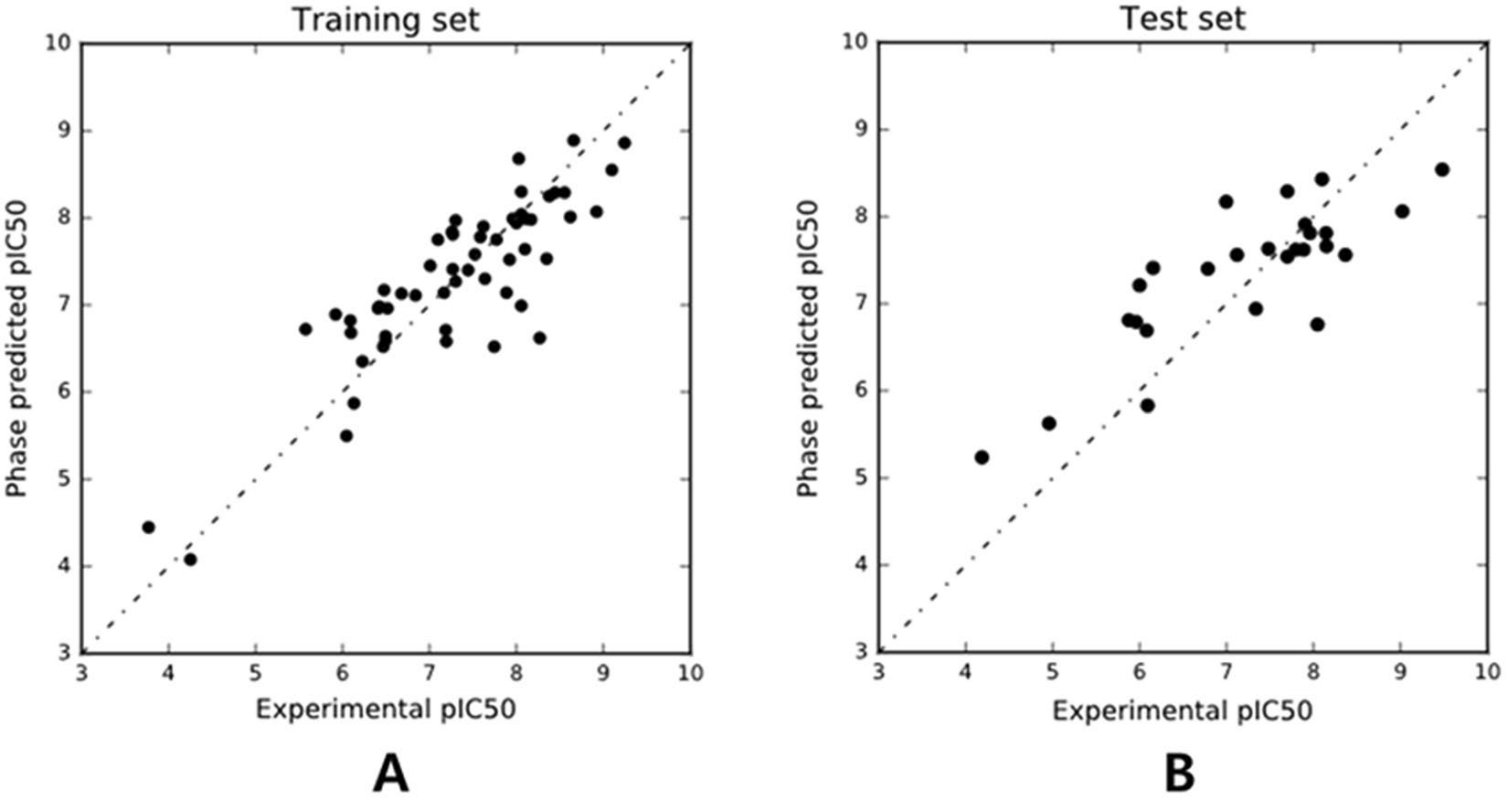
Scatter plot of the observed *versus* predicted activity generated by pharmacophore-based QSAR model (**A**) the training set and (**B**) the test set.

The predictability and validity of AAHPRR.15 common pharmacophore model (test set), based on 55 active compounds (pIC_50_ > 6.9) was judged by the cross validation coefficient (Q^2^ = 0.67) (Table 2). Moreover, the regression coefficient for training set was 0.73, which showed relevance of the model. Further, smaller standard deviation value (SD = 0.56) and root mean square error (RMSE = 0.69) and P value of fourth PLS indicate that the developed AAHPRR.15 model was stable for predicting unknown compounds in the test set. To evaluate the efficacy, model AAHPRR.15 was further validated with the external 27 test set^46,47^. The scatter plots for the experimentally observed and estimated fit values for the training set and the test set molecules is plotted in Fig. 3, while a plot of residual vs. predicted value is shown in Supplementary Fig. 1.

**Table 2:** Means of the statistical variations of the QSAR model.

ID	SD	# Factors	R^2^	P	F	Stability	RMSE	Q^2^	Pearson-R
AAHPRR.10	0.59	1.00	0.7074	9.28E-16	128.1	0.6992	0.7074	0.6592	0.8288
AAHPRR.9	0.59	1.00	0.7074	9.28E-16	128.1	0.6992	0.7074	0.6592	0.8288
AAHPRR.11	0.57	1.00	0.7274	1.40E-16	141.4	0.6063	0.7201	0.6468	0.8266
AAHPRR.15	0.56	1.00	0.7379	4.90E-17	149.2	0.6747	0.6918	0.674	0.8364
AAHPRR.7	0.64	1.00	0.6556	7.21E-14	100.9	0.7685	0.7608	0.6058	0.8099
AAHPRR.8	0.64	1.00	0.6556	7.21E-14	100.9	0.7685	0.7608	0.6058	0.8099
AAHPRR.26	0.58	1.00	0.7186	3.25E-16	135.4	0.7378	0.8339	0.5264	0.7568
AAHPRR.38	0.58	1.00	0.7172	3.73E-16	134.4	0.6295	0.8502	0.5077	0.7276
AAHPRR.24	0.58	1.00	0.7172	3.73E-16	134.4	0.6295	0.8502	0.5077	0.7276
AAHPRR.16	0.62	1.00	0.6783	1.16E-14	111.8	0.6887	0.8311	0.5296	0.7315

The calculated pIC_50_ values of the compounds in the predicted test set and external test set are listed in the Supplementary Table 4. These two plots are important for the predictive potential of QSAR. Residual plots (scatter) were used to detect the existence of outliers from a QSAR model^48,49^. Hence, the developed QSAR model was considered stable and as expected, it was able to endorse the experimental pIC_50_ values for the compounds in the external test set. A predictive correlation coefficient R^2^ value of 0.73 for the external set was obtained for the developed QSAR model. Generally, statistical values of R-square >0.6 and Q-square >0.5 between the predicted and the experimental values renders the model to be good and able to predict the AChE inhibitory activity of compounds not included in the model development process^48–50^.

**Figure 4:**
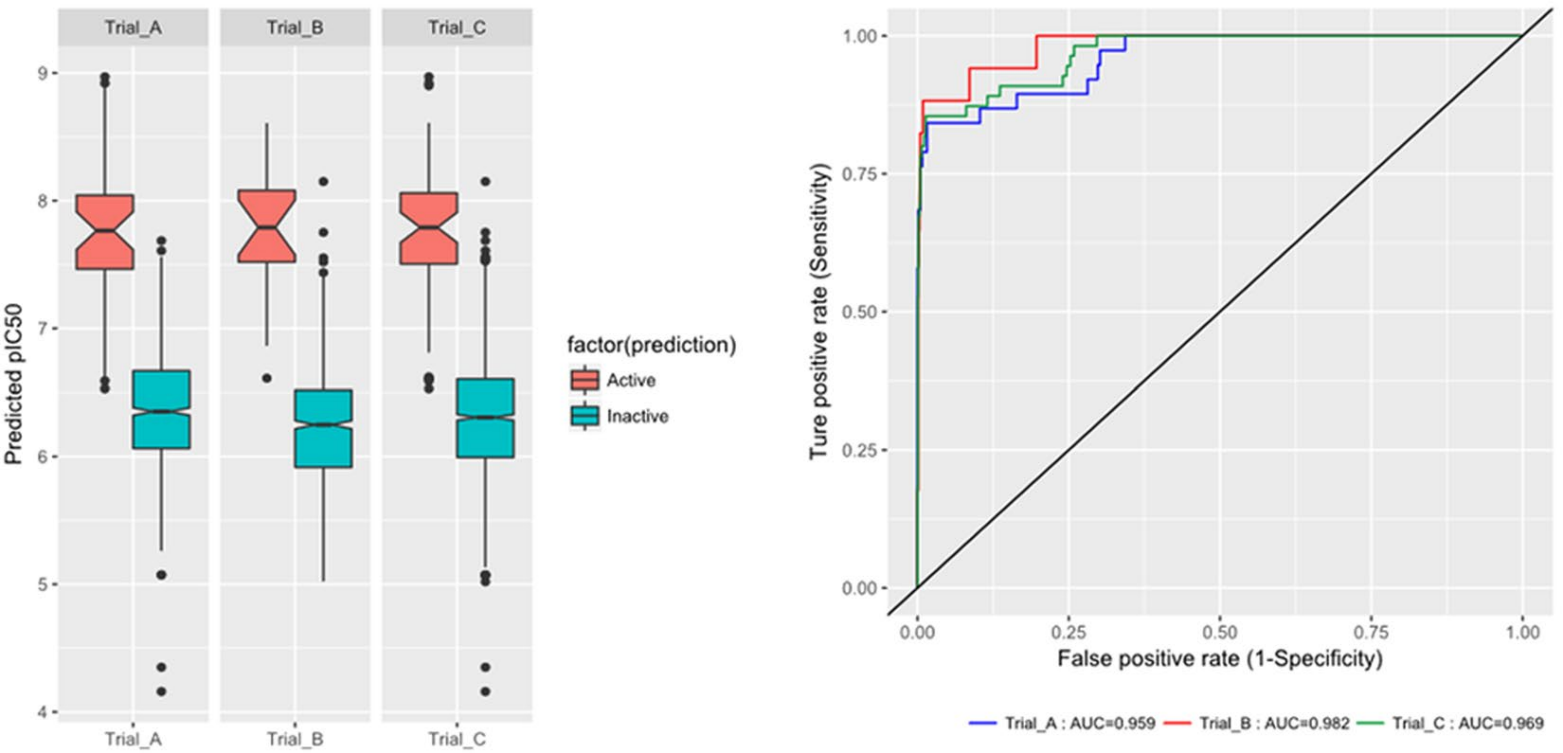
ROC curve obtained by AADRR.6 model against randomly curve.

Moreover, to validate the discriminatory potential, model AAHPRR.15 was tested towards 82 molecules retrieved from the Schrodinger database. The model was able to find 67% of active compounds inside the hit listing. We calculated robust preliminary enhancement (RIE) for the generated models to estimate the contribution of the active compounds ranking in the enrichment. For the hypothesis AAHPRR.15, RIE value was 1.52 (Table 3) indicating the superiority of pharmacophore model ranking over random distribution. Another reliable metrics to evaluate the performance of the pharmacophore model is the AUC of the ROC curve (Fig. 4). The AUC values of three trials showed similar results shown in Fig. 4A, AUC of Trial A (training set and decoy set of training set): 0.959, B (test set and decoy set of test set): 0.982, C(total set and decoy set of total set): 0.969. Stranded criteria to cutoff of AUC is defined as: 0.9 ≤ AUC ≤ 1 is excellent; 0.80 ≤ AUC < 0.9 is good; 0.70 ≤ AUC < 0.8 is fair; 0.50 ≤ AUC < 0.7 is poor; and AUC < 0.5 is a failure^51^. Calculated all AUC means from three trials were excellent and model achieved good value of 0.95 AUC and 0.99 ROC.

**Table 3:** Enrichment data for the generated models.

Pharmacophore model	RIE	ROC	AUC
AAHPRR.7	1.529319	0.908497	0.641403
AAHPRR.8	1.529319	0.908497	0.641403
AAHPRR.9	1.529389	0.95098	0.656109
AAHPRR.10	1.529389	0.95098	0.656109
AAHPRR.11	1.529398	0.947712	0.654977
AAHPRR.15	1.529368	0.931373	0.649321
AAHPRR.16	1.528951	0.852941	0.622172
AAHPRR.24	1.529026	0.895425	0.636878
AAHPRR.26	1.521113	0.862745	0.625566
AAHPRR.38	1.529026	0.895425	0.636878

**Figure 5:**
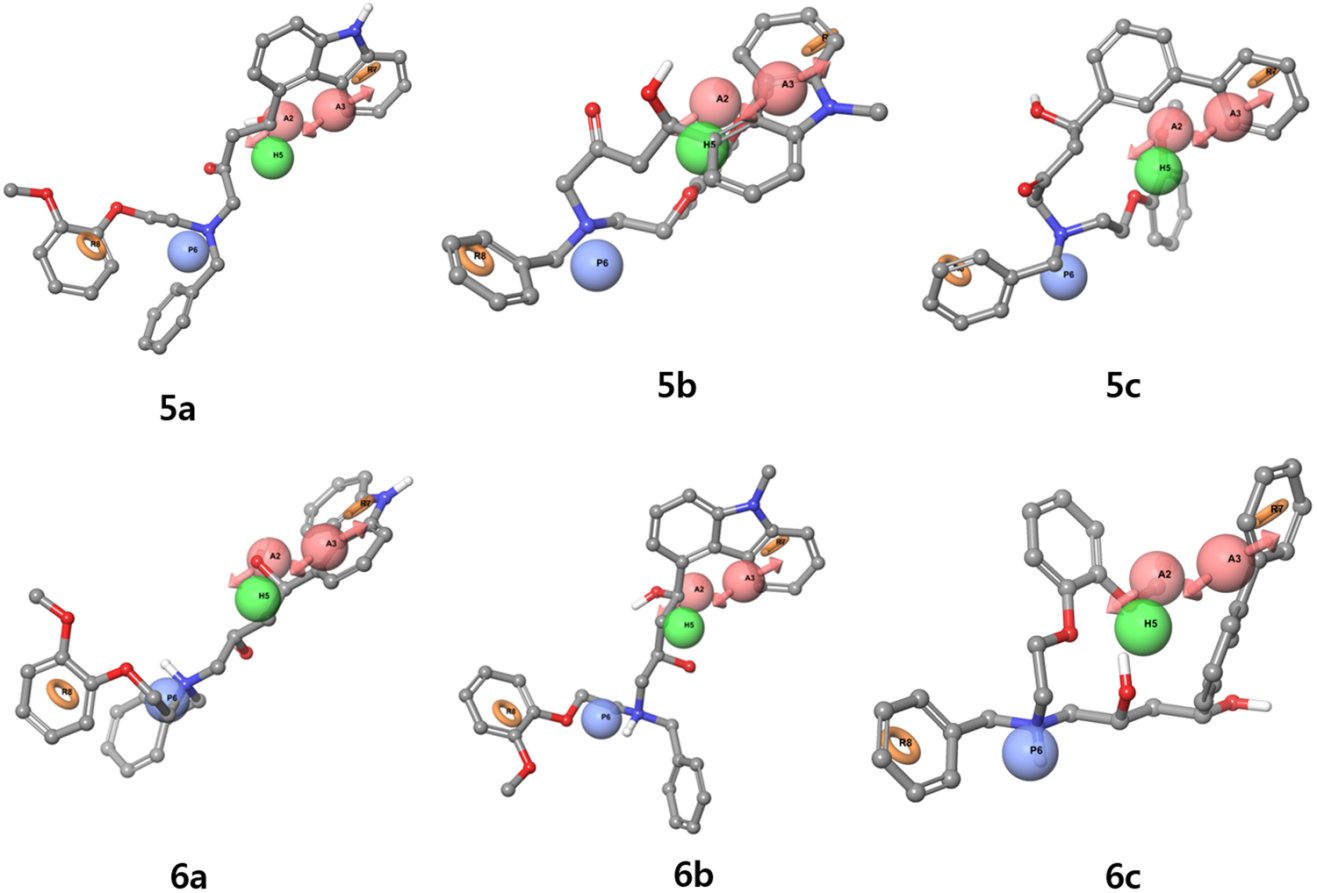
Alignment poses of six compounds (**5a**–**c**, **6a**–**c**) by phase screening using AAHPRR.15 model.

Figure 5 emphasizes on the comparative alignment of the developed pharmacophore model with that to AChE and the important interactions of the AAHPRR.15 model which become further utilized for validating our developed model^52^. Upon superimposition of the pharmacophore model to the docked complex of this series AAHPRR.15, it was observed that both the ligands obtained possessed the same orientation. The distance between each feature in the AAHPRR. 15 model shown in Fig. 2A,B. Figure 5 emphasize the features of catalytic pocket R8 and P6. At first, P6 features showed pi-cation interactions with Tyr337 and Trp86. R8 bind with amino acid residue Trp86 in internal catalytic site and H5 feature in peripheral site did not match any known interaction. Although the other site, H5, did not interact with the receptor, their presence in dataset can serve as a common feature for lead optimization process. Dual inhibitory site was found with a long carbon chains linking in the catalytic and peripheral regions and it affected hydrophobic region in the dataset. The two features, A2 and R7 feature important for peripheral site binding and well matched with the pi-pi interaction of AChE inhibitors with Trp286 and shown the H-bond with Phe295. The other site A3 although not make any interaction with the receptor but due the presence on this dataset can serves as optimization process.

### Pharmacophore based virtual screening

Pharmacophore based virtual screening and docking simulation was performed using the library consisting of commercial compounds and in-house compounds (real/virtual derivatives extended from real scaffold) as in-house 3D-database prepared for virtual screening of any model. Phase screening performed in complex based pharmacophore generation was used as the model to screen the potential AChE inhibitors using Schrödinger software. These molecules were again screened for drug like compounds by applying Lipinski’s rule of five and finally 82 compounds were obtained which might be useful in rational design of new AChE inhibitors. Moreover, docking simulation was performed followed by the protocol of Glide. As a result, 417 compounds with top scores during the pharmacophore based virtual screening and the top 82 from the docking simulation were selected from the list for further structural clustering analysis. Phase screening results are shown in Table 4. The range of fitness scores was from 0.34 to 1.323. The fitness scores of the six compounds were similar, with **6a** having the highest values. The six compounds (Fig. 5) were finally selected from the top ranking compounds sorted by fit value. Predicted activities was measured in logarithmic molar concentration and it was similar to **6a** having the highest value. Most predicted activity values, determined during model validation using ROC curves, were around 5.6, which was regarded as an inactive value. Although predicted activity value was near inactive, there were difference between the in-house compounds and ZINC decoy sets. In this model, polarity and the ring structure were key features for docking at the AChE binding site. Screening compound showed three features, predicted activity pIC_50_ was low because of relatively unimportant acceptor and hydrophobic features. In addition, align scores, vector scores and volume scores were not good because the model was generated based on a docking pose and the dataset was also prepared without any structural correlation.

**Table 4:** AAHPRR.15model phase screening results (PDB ID: 4EY7).

Structure	Compound name	Num Sites Matched	Matched Ligand Sites	Align Score	Fitness	Pred Activity(1)	Vector Score	Volume Score
	7a	4	A(6) A(-) H(10) P(13) R(-) R(17)	1.070	0.797	5.226	0.534	0.193
	7b	4	A(4) A(5) H(-) P(-) R(14) R(16)	1.156	0.864	5.360	0.608	0.232
	7c	4	A(2) A(-) H(-) P(12) R(15) R(14)	1.104	0.801	5.468	0.445	0.304
	7d	4	A(4) A(2) H(7) P(-) R(12) R(-)	1.100	0.774	5.484	0.568	0.151
	7e	4	A(3) A(4) H(-) P(-) R(16) R(18)	1.443	0.749	5.500	0.649	0.240
	7 f	4	A(4) A(1) H(-) P(-) R(15) R(13)	1.563	0.560	5.456	0.544	0.227
	7 g	4	A(1) A(-) H(12) P(15) R(-) R(17)	1.083	0.940	5.488	0.695	0.181
	7 h	4	A(5) A(3) H(-) P(17) R(-) R(20)	0.881	0.989	5.353	0.637	0.184
	7i	4	A(-) A(9) H(-) P(19) R(23) R(20)	1.147	1.023	5.419	0.760	0.234
	7j	4	A(4) A(5) H(-) P(-) R(15) R(18)	1.137	1.074	5.464	0.777	0.262
	7k	4	A(6) A(3) H(-) P(-) R(19) R(15)	0.872	1.025	5.078	0.580	0.272
	7 l	4	A(2) A(4) H(-) P(-) R(17) R(20)	1.287	0.799	5.239	0.636	0.213
	7 m	4	A(4) A(-) H(-) P(13) R(14) R(18)	1.146	0.945	5.374	0.639	0.276
	7n	4	A(3) A(-) H(-) P(13) R(15) R(19)	1.092	0.989	5.365	0.644	0.286
	7o	6	A(3) A(6) H(11) P(16) R(20) R(19)	1.304	0.813	5.319	0.653	0.246
	7p	4	A(1) A(4) H(-) P(-) R(17) R(19)	0.884	1.017	5.369	0.598	0.253
	7q	4	A(1) A(-) H(-) P(12) R(15) R(14)	1.274	0.594	5.473	0.357	0.278
	7r	4	A(3) A(1) H(-) P(11) R(-) R(12)	0.895	1.069	5.425	0.693	0.215
	7 s	4	A(3) A(1) H(-) P(11) R(-) R(13)	0.906	1.061	5.457	0.677	0.229
	7n	4	A(2) A(3) H(-) P(-) R(13) R(16)	1.123	0.880	5.470	0.634	0.204
	7t	5	A(3) A(2) H(-) P(12) R(15) R(14)	1.388	0.877	5.379	0.690	0.319
	7 u	5	A(4) A(2) H(8) P(10) R(14) R(-)	1.529	0.571	5.438	0.652	0.152
	7 v	4	A(6) A(2) H(-) P(-) R(15) R(13)	1.118	1.014	5.268	0.736	0.232
	7w	5	A(1) A(4) H(-) P(12) R(15) R(13)	1.532	0.752	5.372	0.711	0.276
	7 x	4	A(1) A(-) H(11) P(12) R(-) R(13)	1.243	0.917	5.265	0.757	0.185
	7 y	5	A(4) A(1) H(-) P(11) R(14) R(12)	1.727	0.341	5.470	0.501	0.215
	7z	5	A(-) A(3) H(14) P(19) R(23) R(21)	1.558	0.681	4.920	0.712	0.223
	5a	4	A(2) A(-) H(-) P(8) R(9) R(13)	1.207	1.134	5.590	0.828	0.311
	5b	4	A(2) A(-) H(-) P(8) R(10) R(11)	1.305	1.102	6.120	0.893	0.268
	5c	4	A(2) A(-) H(-) P(7) R(9) R(8)	1.368	1.121	5.809	0.918	0.299
	6a	4	A(3) A(-) H(-) P(9) R(13) R(12)	0.994	1.323	5.731	0.810	0.403
	6b	4	A(3) A(-) H(-) P(9) R(13) R(14)	0.559	1.244	5.558	0.633	0.302
	6c	4	A(3) A(-) H(-) P(8) R(10) R(9)	1.330	1.175	5.594	0.923	0.326

### Chemistry

**Figure 6:**
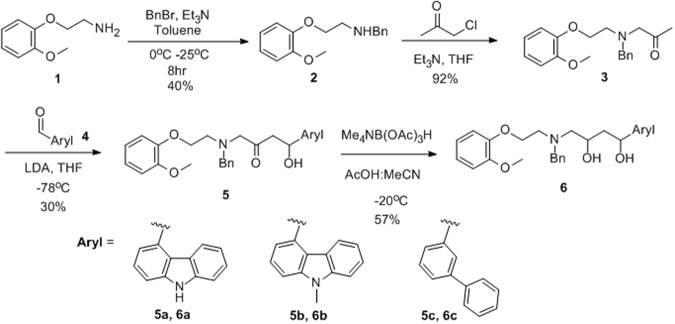
Organic synthetic route of six compounds (**5a**–**5c**, **6a**–**c**).

According to the strategy and design, the predicted compounds **5** and **6** could be synthesized through the synthetic scheme (Fig. 6). The benzylation product **2** from the commercially available starting material **1** could be acquired under reductive amination or direct *N*-alkylation and direct alkylation of benzyl bromide with triethylamine as a base showed better isolation yield (40%) than the yield of reductive amination (17%). The condition of 1-Chloropropane-2-one and triethylamine provided three carbon elongation with the compound **2** to produce the compound **3** with the yield of 92%. In sequence, direct aldol reaction between the compound **3** and aldehyde **4** generated the racemic beta hydroxyl ketone products **5a**, **5b** and **5c** with up to 30% yield. In addition, the mild reaction using tetramethylammonium triacetoxyborohydride (Me_4_NB(OAc)_3_H) produced racemic 1,3-diol compounds **6a**, **6b** and **6c** (up to 57% yield).

### Molecular docking analysis

**Figure 7:**
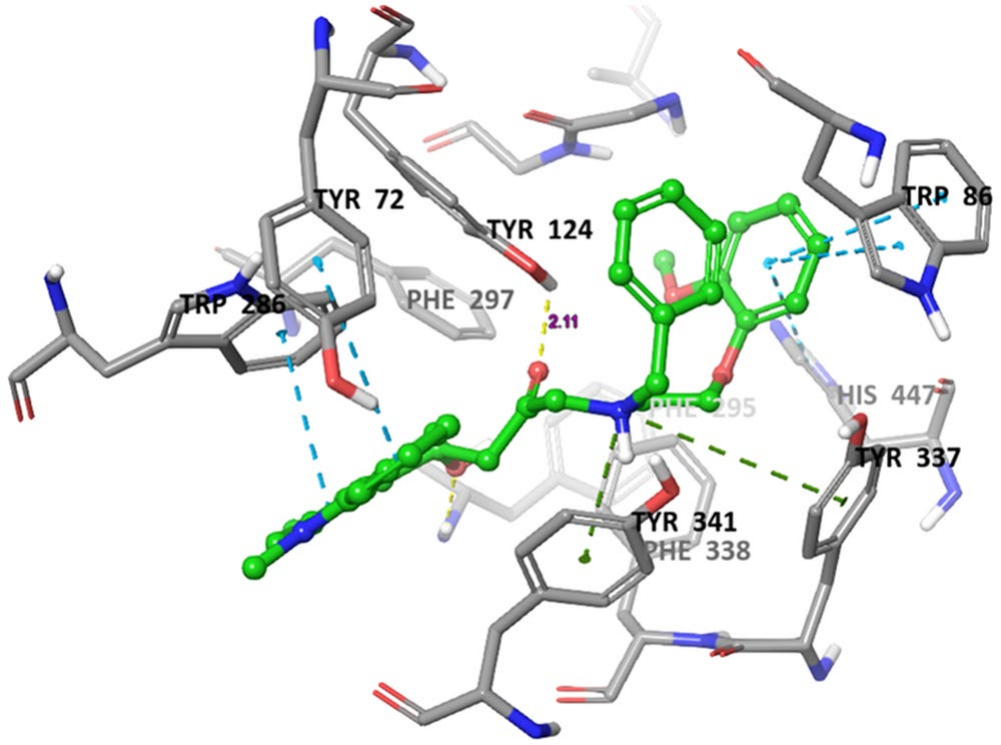
Binding mode of compound **5b** interaction in the catalytic and peripheral pocket of 4EY7. The interactions are depicted with different colors: pi-pi (blue dotted line), hydrogen bond (yellow dotted line) and pi-cation (green dotted line).

**Figure 8:**
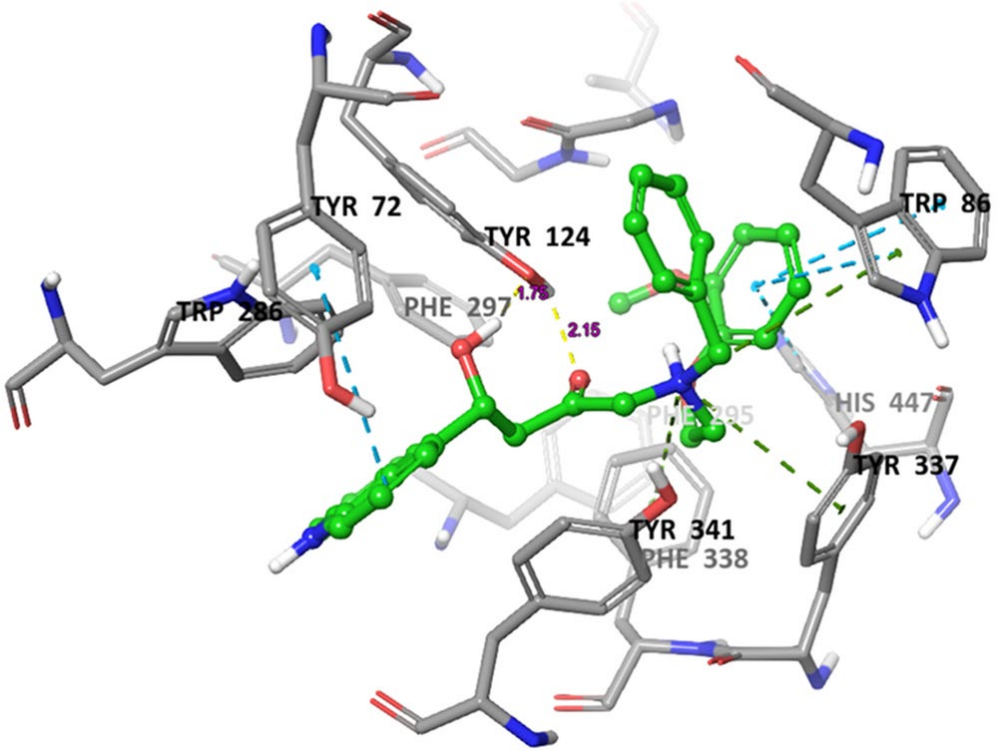
Binding mode of compound **5a** interaction in the catalytic and peripheral pocket of 4EY7. The interactions are depicted with different colors: pi-pi (blue dotted line), hydrogen bond (yellow dotted line) and pi-cation (green dotted line).

**Figure 9:**
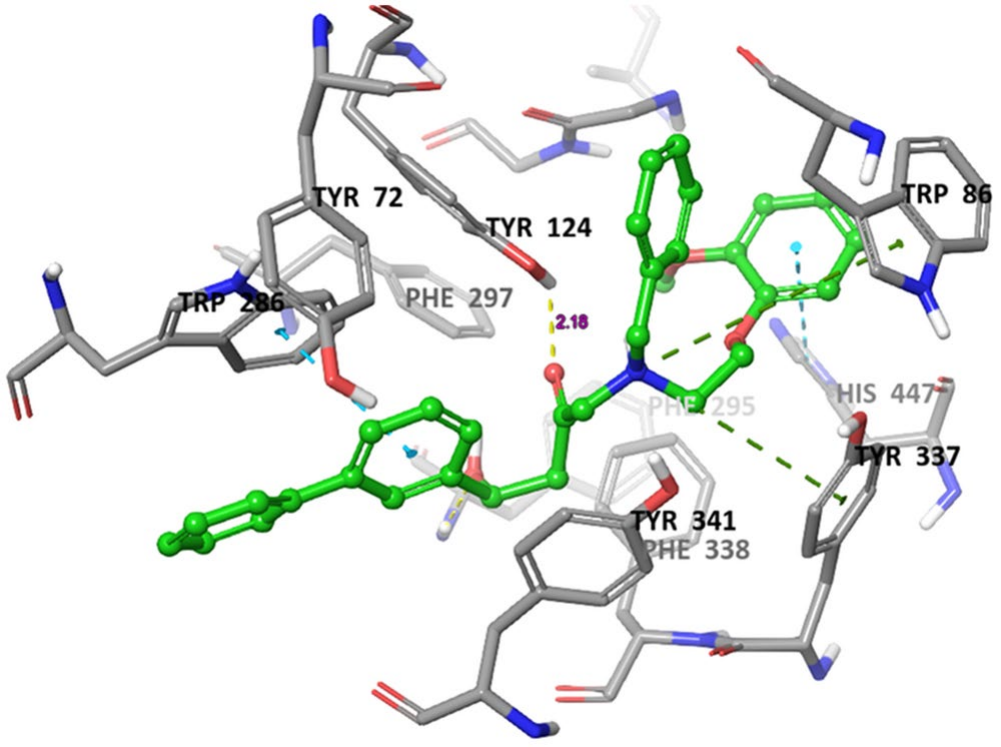
Binding mode of compound **5c** interaction in the catalytic and peripheral pocket of 4EY7. The interactions are depicted with different colors: pi-pi (blue dotted line), hydrogen bond (yellow dotted line) and pi-cation (green dotted line).

**Figure 10:**
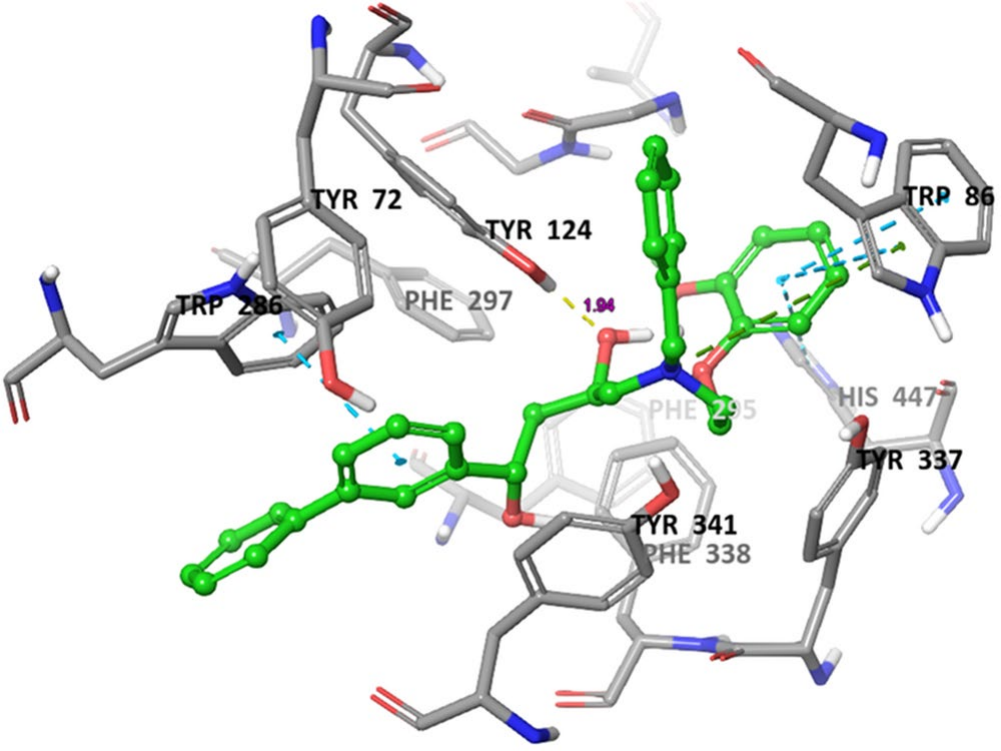
Binding mode of compound **6c** interaction in the catalytic and peripheral pocket of 4EY7. The interactions are depicted with different colors: pi-pi (blue dotted line), hydrogen bond (yellow dotted line) and pi-cation (green dotted line).

**Figure 11:**
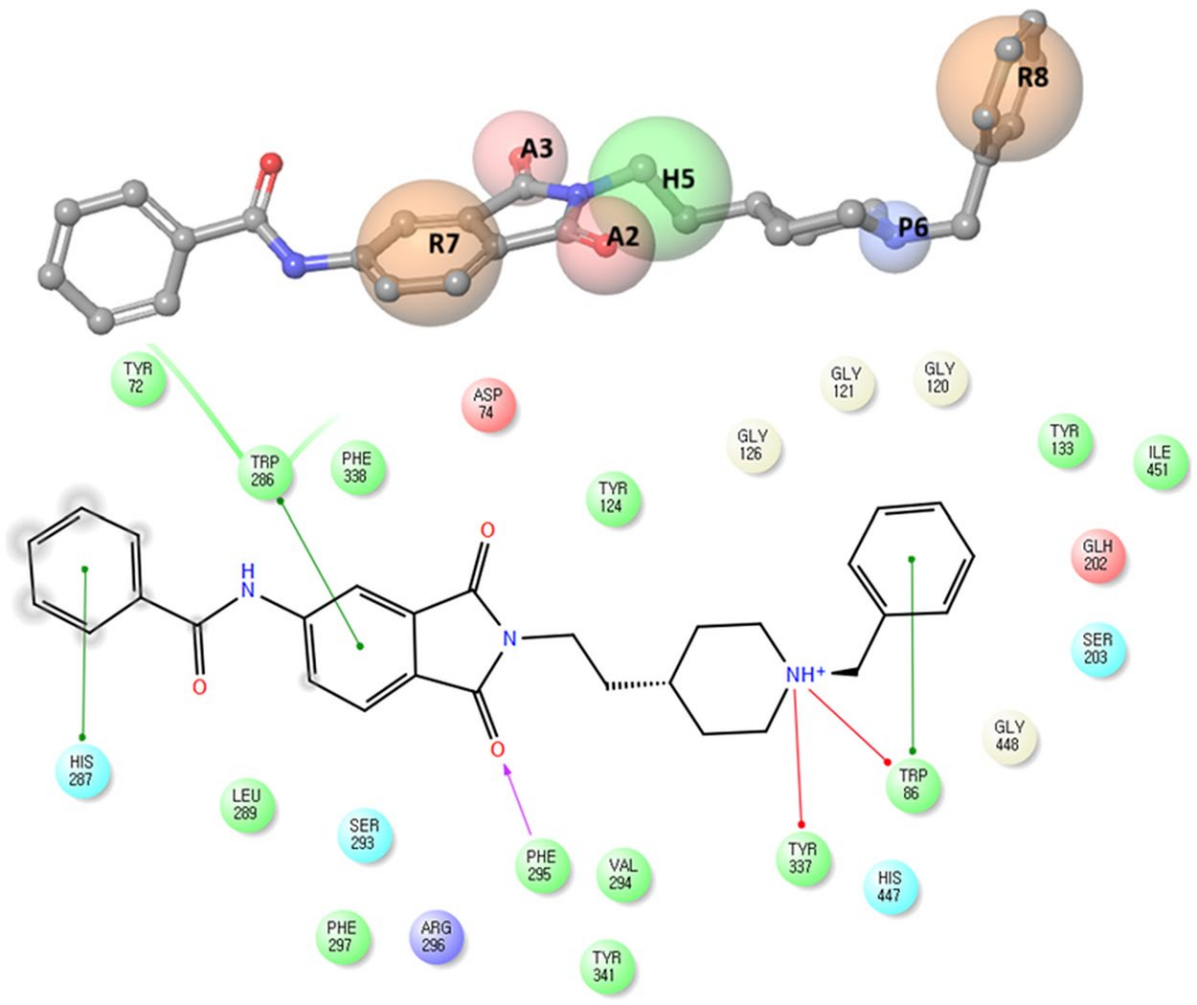
Docking result of a reference ligand and near residue information which is used to build pharmacophore model AAHPRR.15 in 4EY7. The interactions are depicted with different colors: pi-pi (green line), hydrogen bond (violet line) and pi-cation (red line).

Molecular docking is a crucial tool for exploring the interactions between the target protein and a small molecule. In order to find out the structure and activity relationships of the new AChE inhibitors, molecular docking was performed in same parameter settings using the Glide program. All of the compounds were proposed to bind with AChE within the active pocket. As the most representative sample, the binding mode of AChE (PDB ID: 4EY7) was chosen to proceed with further structure activity relationship (SAR) analysis. The role of water as a ligand is very important in both increasing and decreasing the binding energy of a drug^52^. Athough it was reported that waters near to interactive residues (F295, Y121, Y341 and Y337) in 4EY7 mediated the piperidine ring flip of donepezil or hydrogen bond to induce the tight binding, the position of the waters in the x-ray can be of limited use for energetics of donepezil derivatives or their similar scaffolds resulting from core hopping of donepezil. To consider hit compounds differentiating with donepezil, the waters in the binding site were deleted. The proposed binding modes of the top four scorers (**5a**, **5b**, **5c** and **6c**) are illustrated in Figs 7–10. The orientation of each of these ligands resembles that of the native ligand, donepezil. The proposed binding was thus very similar to that of donepezil in the crystallized structure. The interactions were dominated in the region of His447 and Trp86 amino acid residues due to pronouncing existence of the pi-cation interaction at the catalytic anionic site and the hydrogen bonds with Tyr124 and Phe295 at the edge of the peripheral site region (Fig. 11). Compound **5b** against the AD target protein AChE showed a high binding affinity docking score indicated by a docking score of −16.176 and forms a H-bond of length 2.1 Å to the polar aliphatic residue that is, His447 and Trp86. The other site A2 and A3 form two H-band of length 1.7 and 2.1 Å to the hydrophobic residue Phe295 and Tyr124. Our Glide XP-docking result also exposed π–π stacking, the site P6 was interacting with the receptor and form pi-pi stacking with two amino acid residues Tyr341 and Tyr337 and form a salt bridge with Asp74. Thus, the functionalities such so positively charged groups at P6 and aromatic rings R8 and R7 were identified to be important for AChE opening activity shown in Fig. 2. Even though site H5 although not interacting with the receptor but due to their presence in dataset can serve as targets for lead optimization process.

On the other hand, docking results for **5a**, **5c** and **6c** against the target protein AChE showed a high binding affinity docking score of −11.604, −16.147 and −14.797 and forms a H-bond of length 1.7, 2.0, 2.1 and 1.9 Å to the hydrophobic residue that is, Tyrosine-124 and Phenylalanine-295. Favorable hydrogen bond interactions between the ligand and the protein residues were encountered at the binding site. Both **5a** and **6a** form four hydrogen bonds with the protein residues, viz. Tyr124 (Figs 8–10 and Table 5). Moreover, the hydrogen bond with Phe295 is hydrophobically packed, imparting additional reward to **5b** and **5c**. The third best scorer, L23, displays only two hydrogen bonds (Lys254 and Lys352). In spite of higher hydrogen bond contribution as compared to the top three scorers, the overall score of **6a** is low due to other influential factors such as low lipo and EvdW values. On the other hand, docking results of compound **5a**, **5c** and **6c** shown the π–π stacking of R8pharmacophoric feature with HIS-447, TRP-86, TYR-341, TYR-72 and R7 with TYR-341 and TYR-72 (Supplementary Figs 2–4) residues, an attractive and noncovalent interactions between aromatic rings and play an important role in stabilization of inhibitor at the active site.

**Table 5:** The total number and sites of hydrogen bonds and Pi-Pi stacking formed between the ligands and the protein residues at the donepezil binding domain.

Compound Name	Docking Score	Amino acid involved in active pocket in 4 Ă	Involved group of Amino Acid	Length of H-bond Ă	No. of H-bond Bond	Pi-Pi stacking	Length of Pi-Pi stacking Ă	Pi-cation interaction	Length of Pi-cation interaction Ă	Salt bridge interaction
5a	−11.604	SER-203, HIS-447, GLY-120, GLY-121, GLH-202, ASN-87, GLY-126, THR-83, SER-125, PHE-338, TYR-341, TYR-124, PHE-297, ARG-296, VAL-294, SER-293, LEU-289, TRP-286, THR-75, LEU-76, TYR-72, TYR-337, ASP-74, TRP-86, PHE-295	TYR-124	1.75 2.16	2	HIS-447 TRP-86 TYR-341 TYR-72	5.05 5.07 4.67 5.42	TRP-86 PHE-338 TYR-337	5.80 6.07 4.60	ASP-74
5b	−16.176	ALA-204, GLY-120, GLY-122, SER-203, HIS-447, TRP-86, PHE-338, SER-125, TYR-341, TYR-337, ASP-74, PHE-295, TRP-286, PHE-297, ARG-296, VAL-294, LEU-289, SER-293, TYR-72, LEU-76, THR-75, TYR-124, GLY-448, TYR-133, GLH-202, GLY-121	PHE-295 TYR-124	2.03 2.11	2	HIS-447 TRP-86 TRP-72 TRP-286	4.84 5.20 5.24 4.32	TYR-337 TYR-341	5.43 3.60	ASP-74
5c	−16.147	ASN-87, SER-125, GLY-126, GLY-122, GLY-121, HIS-447, SER-203, GLY-120, TYR-133, ILE-451, GLH-202, GLY-448, PHE-295, PHE-297, ASP-74, VAL-294, ARG-296, TRP-286, TYR-72, SER-293, LEU-289, TYR-337, PHE-338, TYR-341, TYR-124, TRP-86	TYR-124 PHE-295	2.18 2.19	2	HIS-447 TRP-286	4.78 4.06	TYR-337 TRP-86	5.52 6.54	ASP-74
6a	−9.275	SER-125, LEU-130, TYR-133, GLY-448, GLY-120, ILE-451, GLH-202, GLY-121, GLY-122, ALA-204, SER-203, HIS-447, TYR-337, PHE-338, PHE-295, TYR-341, TYR-72, ASP-74, TYR-124, PHE-297, VAL-294, LEU-289, ARG-296, SER-293, TRP-286, LEU-76, THR-75, TRP-86, GLY-126	TYR-124	1.90	1	HIS-447 TRP-86 TYR-341 TYR-72	4.78 4.37 4.85 5.42	TRP-86 TYR-341	6.11 4.55	
6b	−14.439	TYR-72, HIS-447, PHE-205, TYR-124, GLY-122, SER-203, SER-125, GLY-120, GLY-121, TYR-133, GLY-126, TRP-86, GLH-202, GLY-448, THR-83, TYR-337, ILE-451, PHE-338, TYR-341, ASP-74, PHE-297, TRP-286, ARG-296, SER-293, VAL-294, LEU-289			0	HIS-447 PHE-338 TRP-86 TRP-286	5.26 5.14 4.27 4.14 4.36	TYR-337 PHE-338 TYR-341	4.68 5.27 4.08	ASP-74
6c	−14.797	GLU-292, SER-293, ARG-296, LEU-289, TYR-72, TYR-124, PHE-338, THR-83, GLY-126, ASN-87, PRO-88, SER-125, GLY-121, GLY-122, ALA-204, SER-203, HIS-447, GLY-120, ILE-451, TYR-337, GLY-448, GLH-202, ASP-74, TRP-86, PHE-297, PHE-295, TYR-341, VAL-294, TRP-286	TYR-124	1.94	1	HIS-447 TRP-86 TRP-286	4.64 5.07 4.01	TRP-86 PHE-338	5.83 6.49	ASP-74

### Compliance with pharmacokinetic and toxicity risks assessment

The Pharmacokinetic properties (ADMET) are of prime importance for a molecule eligible to be an active drug *in vivo*. During clinical trials, poor ADMET properties often lead to failure of an otherwise potent drug. Thus, it is crucial to investigate the pharmacokinetic profile of a potential drug as well. A poor absorption or permeation is more likely when a ligand violates Lipinski’s rule of five^29^, i.e. it has more than 5 hydrogen bond donors and 10 hydrogen bond acceptors, its molecular weight is over 500, the logP (n-octanol and water partition coefficient) is over 5 and the sum of N’s and O’s is over 10. This rule is the most widespread method to assess the ‘drug-like’ properties of molecules during the early stages of the drug discovery process^30,31,42^. Therefore, in order to evaluate the druggability of in house compounds using Lipinski’s rule of five, the physically important descriptors and pharmaceutically relevant ADMET properties were evaluated using the QikProp4 module. We found that all the compounds violate the Lipinski’s rule of five due to high MW (greater than 500 Da.) except **5c**, therefore showed low solubility and low cell membrane permeability. Rest of the active derivatives follow Lipinski’s rule and have reliable polarity for better permeation and absorption as revealed by H-bond donors and H-bond acceptors (Table 6).

**Table 6:** Compliance of active compound with computational parameters of drug likeness and ADME properties.

Compound	MW	log P	donorHB	accptHB	^#^NandO	PSA
5a	508.616	6.042	1	6.2	6	73.002
5b	522.643	6.619	0	6.2	6	64.701
5c	495.617	6.881	0	6.2	5	62.494
6a	510.632	5.37	3	6.9	6	65.447
6b	524.658	6.047	2	6.9	6	54.064
6c	497.633	6.227	2	6.9	5	51.733

Note: MW, Molecular weight; Log P, Octanol/water partition coefficient; donorHB, Hydrogen bond donor; accptHB, Hydrogen bond acceptor; ^#^NandO, Number of nitrogen and oxygen atoms.

Solubility, CNS, Blood brain barrier, partition coefficient, H-band donor and acceptor were calculated for pharmacokinetic property while for toxicity study, mutagenicity, tumorigenicity, irritation effect and risk of reproductive effect were predicted. The toxicity risk predictor locates fragments within a molecule, which indicate a potential toxicity risk. Toxicity screening results showed that compounds all the potential leads pose no risk of mutagenicity, tumorigenicity irritation and reproductive toxicity shown in Table 7. To judge the leads overall potential to qualify for a drug, we calculated overall drug score, which combines drug-likeness, hydrophilicity (cLogP), aqueous solubility (LogS), MW and toxicity risk parameters. The logP value was predicted to determine hydrophilicity of all compounds. This study suggested that predicted log S; log P, BBB, CNS activity and TPSA values of the studied compounds within the acceptable limit (Table 8). The results of toxicity risk assessment screening showed an overall drug score of predicted active compounds. This result further encourages us to discover newer AChE inhibitors for the Alzheimer’s disease.

**Table 7:** Compliance of active compounds with computational toxicity risk parameters.

Compound	MUT	TUMO	IRRI	REP	Drug likeness	Drug-Score
5a	No risk	No risk	No risk	No risk	6.28	0.40
5b	No risk	No risk	No risk	No risk	6.76	0.41
5c	No risk	No risk	No risk	No risk	5.66	0.39
6a	No risk	No risk	No risk	No risk	6.14	0.40
6b	No risk	No risk	No risk	No risk	6.64	0.41
6c	No risk	No risk	No risk	No risk	5.50	0.39

Note: MUT, mutagenicity; TUMO, tumorogenicity; IRRI, irritation; REP, reproduction;

**Table 8:** ADME and pharmacological parameters prediction for active compound using QikProp.

Compound name	log S for aqueous solubility	log Khsa for serum protein binding	log BB for brain/blood	No. of metabolic reactions	Predicted CNS activity	log HERG for K + Channel blockage	Apparent Caco-2 permeability (nm/s)	Apparent MDCK permeability (nm/s)	log Kp for skin permeability	% Human Oral Absorption in GI (+−20%)
5a	−5.258	1.071	−0.61	8	1	−8.01	453.50	232.829	−1.82	83.954
5b	−5.769	1.166	−0.51	8	1	−8.22	593.86	311.611	−1.64	89.429
5c	−6.746	1.183	−0.70	7	1	−9.56	561.59	293.351	−1.15	100
6a	−4.341	0.784	−0.64	7	0	−7.59	452.06	232.031	−1.84	79.993
6b	−4.932	1.000	−0.41	7	1	−7.59	769.19	412.141	−1.48	88.091
6c	−5.482	1.003	−0.61	6	1	−8.53	639.72	337.702	−1.22	100
Stand. range*	(−6.5/0.5)	(−1.5/1.5)	(−3.0/1.2)	(1.0/8.0)	−2 (inactive) +2 active)	(concern below 5)	(<25 poor, >500 great)	(<25 poor, >500 great)	(8.0 to 1.0, Kp in cm/h)	(<25% is poor)

Note: **For 95% of known drugs based on Schrödinger, USA-Qikprop v3.2 (2015) software results.

### *In vitro* AChE inhibitory activity & structure-activity relationship

The structural construction (2-ethyl aryl substituted vicinal amino alcohol) of our in-house compounds **5a**–**c** and **6a**–**c** could not only show compatibility with the optimal pharmacophore model, ‘AAHPRR.15’ but also show unique structure far from cyclic ammonium like piperidinum (donepezil and dataset). To prove the prediction of the synthesized compounds **5a**–**c** and **6a**–**c** under the ‘AAHPRR.15’ model, the compounds **5a**–**c** and **6a**–**c** were evaluated for their inhibition activity toward *AChE*. Concurrently, the *AChE* inhibition activity of tacrine was also carried out to crosscheck as a standard inhibitor. The results of all compounds were showed statistically significantly inhibition activity over *AChE* comparable with negative control group (Supplementary Fig. 6).

**Figure 12:**
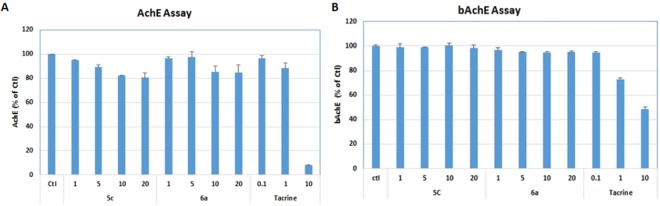
Percentage inhibitory activities of compounds **5a**–**c** and **6a**–**c** in *eeAChE* and horse BuChE.

Less amount of acetylcholine (Ach) with higher amount of acetylcholinesterase (AchE) is responsible for cholinergic abnormalities and subsequently behavioral and cognitive decline with aggravated pathology in dementia Alzheimer’s disease patient^53^. Inhibition of either AchE or BchE selectively or non-selectively can improve the condition of patient with dementia and AD^54^. The synthetic analogues have only been tested *in vitro* at this time, but it is an area that can help future treatment options for AD. In this study we have synthesized six compounds on the basis of structural docking with the belief that it can inhibit the acetylcholinesterase (*AchE*) activity. Among 6 tested compounds, compound **5c** and **6a** presented more distinct concentration-dependency than others so that compound **5c** and **6a** were chosen for further assay getting IC_50_ and selectivity between *AchE* and *BchE* enzyme activity for those compounds. The selected **5c** and **6a** for their inhibitory effect against acetylcholinesterase and butylcholinesterase enzyme activity through modified Ellmen’s method (Fig. 12). Though inhibition of both enzyme activity is required for the proper biological activity for the treatment of Dementia and Alzheimer’s disease like conditions however, some compound has specific role for particular one enzyme while some have nonspecific one and they can inhibit both of the enzymes. Here it is observed that **5c** and **6a** has a selective AchE inhibition with the IC_50_ value 58.33 and 66.05 µM respectively (Table 9) while Tacrine as a positive control has the IC_50_ 5.24 µM (Fig. 12B). Both of the compounds did not showed the effect against BchE. It was the expected result from our molecular docking expectation. Tacrine has been used as positive control for both AchE and BchE inhibition so it showed the IC_50_ about 9.46 µM for BchE inhibition (Fig. 12B). The significant inhibition of AchE and BchE with the treatment of Tacrine in the experiment supports the fact of previous studies about Tacrine as a non-selective choline esterase inhibitor^54,55^. Tacrine was the first drug approved for treatment of AD in 1993^56^. It is a potent inhibitor of both AChE and BuChE. Tacrine was approved both because of efficacy on the ADAS-Cog and on the global measure compared to placebo in phase II and phase III clinical trials of AD subjects^57^. Although our compound has higher value of IC_50_ against AchE, they have the selectivity towards AchE only. Further consideration on the structure modification on these compounds might produce more effective and selective AchE inhibitor in future.

**Table 9:** IC_50_ concentration for Acetylcholinesterase (AchE) and butyrylcholinesterase (bAchE) inhibition by 5c and 6a.

Compound Name	AchE IC_50_(µM)	bAchE IC_50_(µM)
5c	58.33	>500
6a	66.05	>500
Tacrine	5.24	9.46
Tacrine (Ellman method)	0.5	0.058

Experiment was performed according to the Ellman’smethos. Compound treatment was performed for four (1, 5, 10 and 20 µM) concentrations and the final inhibitory concentration for the 50% inhibition of AchE and bAchE was evaluated. Our compounds did not show the impressive effect to inhibit any of the assay.

## Material and Methods

### Chemistry

#### Synthesis of benzyl-[2-(2-methoxy-phenoxy)-ethyl]-amine (compound 2)

Triethylamine (6051 mg, 59.80 mmol) was added to a solution of benzyl bromide (4100 mg, 23.92 mmol) in toluene (15 mL) and cooled to 0 °C. A solution of compound **1** (5000 mg, 29.90 mmol) in toluene (10 mL) was added slowly at the same temperature and allowed to reach 25 °C. The reaction mixture was maintained at room temperature for 8 h. Water (25 mL) was added to the reaction mass and stirred for 15 min. The layers were separated and the organic layer was washed with water (15 mL) and dried over sodium sulfate. The solvent was distilled off completely under reduced pressure to obtain benzyl-[2-(2-methoxy-phenoxy)-ethyl]-amine 2 as residue. The concentrated crude product was purified by column chromatography (silica gel, 15–80% gradient EtOAc/Hex) to obtain the known compound **2** as colorless oil and identified with already reported spectra data.

#### Synthesis of 1-{benzyl-[2-(2-methoxy-phenoxy)-ethyl]-amino}-propan-2-one (compound 3)

A mixture of compound 2 (2400 mg, 9.32 mmol), 1-chloropropane-2-one (1790 mg, 18.74 mmol) and Et_3_N in THF (15 mL) was stirred at rt for 15 hrs and filtered. The filtrate was diluted with EtOAc (80 mL), washed with saturated aq. NaHCO_3,_ water, brine dried over anhydrous magnesium sulfate and concentrated under reduced pressure to afford crude compound. The crude product was purified by column chromatography (silica gel, 15–80% gradient EtOAc/Hex) to obtain the compound **3** as yellow oil.

^1^H NMR (600 MHz, CDCl_3_) δ 7.37 (dd, *J* = 7.9, 0.9 Hz, 2H), 7.34–7.29 (m, 2H), 7.28–7.23 (m, 1H), 6.94–6.83 (m, 4H), 4.11 (t, *J* = 5.7 Hz, 2H), 3.83 (s, 3H), 3.81 (s, 2H), 3.48 (s, 2H), 3.04 (t, *J* = 5.7 Hz, 2H), 2.12 (s, 3H); ^13^C NMR (150 MHz, CDCl_3_) δ 208.92, 149.48, 148.22, 138.81, 128.95 (2C), 128.37(2C), 127.26, 121.23, 120.84, 113.21, 111.77, 67.81, 64.59, 59.61, 55.77, 53.02, 27.55 ppm; HRMS-ESI (m/z): Calculated for C_19_H_23_NO_3_ M^+^: 313.1680, Found: 313.1678.

#### Synthesis of compound **5a**, **5b** and **5c**

The LDA solution 1.0 M in THF (133.4 mg, 1.24 mmol) was cooled to −78 °C for 5–10 minutes. A solution of compound **3** (300 mg, 0.95 mmol) in 1.5 mL of THF was added dropwise. After being stirred for 20 min, arylaldehyde (0.95 mmol) was added rapidly. The reaction mixture was quenched with dilute solution of NH_4_Cl (pH 4–5) after 1 hr and then partitioned between H_2_O and EtOAc. The organic phase was washed with NH_4_Cl solution. Flash chromatography (ethyl acetate/hexane 1~15%, SiO_2_ deactivated with NH_4_OH drops) of the residue obtained after drying (Na_2_SO_3_) and evaporation gave product **5a**, **5b and 5c**.

#### 1-(benzyl(2-(2-methoxyphenoxy)ethyl)amino)-4-(9H-carbazol-4-yl)-4-hydroxybutan-2-one (**5a**)

^1^H NMR (600 MHz, Acetone) δ 10.47 (s, 1H), 8.29 (d, J = 8.0 Hz, 1H), 7.54 (d, J = 8.1 Hz, 1H), 7.46–7.37 (m, 6H), 7.28 (dd, J = 10.6, 4.2 Hz, 2H), 7.25–7.21 (m, 1H), 7.18 (ddd, J = 8.1, 7.2, 1.0 Hz, 1H), 6.91–6.81 (m, 4H), 4.73 (d, J = 26.7 Hz, 1H), 4.64 (bs, 1H), 4.13–4.08 (m, 2H), 3.94–3.84 (m, 3H), 3.68 (s, 3H), 3.16 (dd, J = 15.9, 9.9 Hz, 1H), 3.04 (t, J = 5.8 Hz, 2H), 2.92 (dd, J = 15.9, 2.8 Hz, 1H), 2.84 (d, J = 16.0 Hz, 1H); 13C NMR (150 MHz, Acetone) δ 208.99, 149.74, 148.63, 140.50, 140.23, 140.14, 139.36, 128.97, 128.09, 128.06, 126.86, 125.35, 125.06, 123.28, 122.14, 121.61, 120.93, 120.63, 118.94, 115.27, 113.45, 112.09, 110.66, 110.61, 109.57, 109.51, 67.66, 64.79, 59.06, 55.04, 52.51, 47.58 ppm; HRMS-ESI (m/z): Calculated for C_19_H_23_NO_3_ M^+^: 508.2362, Found: 508.2364.

#### 1-(benzyl(2-(2-methoxyphenoxy)ethyl)amino)-4-hydroxy-4-(9-methyl-9H-carbazol-4-yl)butan-2-one (**5b**)

^1^H NMR (600 MHz, Acetone) δ 8.22 (d, *J* = 7.9 Hz, 1H), 7.56 (d, *J* = 8.2 Hz, 1H), 7.51–7.43 (m, 4H), 7.36–7.31 (m, 3H), 7.28–7.22 (m, 2H), 6.97–6.82 (m, 5H), 5.27 (d, *J* = 6.4 Hz, 1H), 4.11 (t, *J* = 5.6 Hz, 3H), 3.93 (s, 2H), 3.85 (s, 3H), 3.81 (s, 4H), 3.52 (s, 2H), 2.99 (t, *J* = 5.6 Hz, 3H); ^13^C NMR (150 MHz, Acetone) δ 209.01, 149.77, 148.65, 141.23, 141.08, 140.63, 139.37, 128.99, 128.10, 127.99, 126.90, 126.56, 125.48, 125.19, 123.35, 121.75, 120.97, 120.67, 118.96, 118.30, 115.38, 113.48, 112.12, 108.60, 107.52, 67.97, 67.69, 64.83, 59.10, 55.09, 52.55, 47.38, 28.43 ppm; HRMS-ESI (m/z): Calculated for C_19_H_23_NO_3_ M^+^: 522.2518, Found: 522.2519.

#### 4-([1,1′-biphenyl]-3-yl)-1-(benzyl(2-(2-methoxyphenoxy)ethyl)amino)-4-hydroxybutan-2-one (**5c**)

^1^H NMR (600 MHz, CDCl_3_) δ 7.62–7.55 (m, 4H), 7.46 (t, *J* = 7.7 Hz, 3H), 7.41–7.33 (m, 7H), 6.98–6.85 (m, 4H), 5.13 (dd, *J* = 8.7, 3.7 Hz, 1H), 4.15 (t, *J* = 5.0 Hz, 2H), 3.90–3.81 (m, 2H), 3.79 (s, 3H), 3.56 (s, 2H), 3.08 (t, *J* = 5.4 Hz, 2H), 2.94–2.92 (m, 2H);^13^C NMR (150 MHz, CDCl_3_) δ 210.97, 149.21, 147.91, 141.90, 140.61, 140.25, 138.19, 137.61, 128.90, 128.58(2C), 128.27(2C), 127.23, 127.10, 127.01(2C), 126.88(2C), 125.91(2C), 121.17, 120.68, 113.03, 111.58, 69.59, 67.60, 64.59, 59.72, 55.51, 53.07, 48.46 ppm; HRMS-ESI (m/z): Calculated for C_32_H_33_NO_4_ M^+^: 495.2410, Found: 495.2410.

#### Synthesis of compounds **6a**, **6b** and **6c**

The solution of tetramethylammonium triacetoxyborohydride (206 mg, 0.08 mmol) in anhydrous acetic acid (0.5 mL) and anhydrous acetonitrile under Ar atmosphere was stirred at −50 °C. At the temperature, the solution of compound **5** (50 mg, 0.01 mmol) in anhydrous acetonitrile was transferred into the cooled mixture through cannula and warmed into −20 °C. After termination of the reaction during ca. 20 hours at −20 °C, the reaction mixture was quenched with aqueous Rochelle salt solution and extracted with methylenechloride more than 3 times. The organic layers were combined, dried over magnesium sulfate and filtered and the filtrate was concentrated in vacuo to produce the crude mixture. The concentrated crude product was purified by column chromatography (silica gel, 15–50% gradient EtOAc/Hex) to obtain the compound **6** as a pale yellow.

#### 4-(benzyl(2-(2-methoxyphenoxy)ethyl)amino)-1-(9H-carbazol-4-yl)butane-1,3-diol (**6a**)

^1^H NMR (600 MHz, Acetone) δ 10.39 (s, 1H), 8.52 (d, *J* = 8.0 Hz, 1H), 7.52 (d, *J* = 8.0 Hz, 1H), 7.48 (ddd, *J* = 6.3, 2.0, 0.7 Hz, 1H), 7.43–7.35 (m, 5H), 7.28 (dd, *J* = 10.2, 4.6 Hz, 2H), 7.22 (t, *J* = 7.3 Hz, 1H), 7.13 (ddd, *J* = 8.1, 7.2, 1.0 Hz, 1H), 6.96 (dd, *J* = 8.3, 1.5 Hz, 1H), 6.93–6.89 (m, 3H), 6.88–6.84 (m, 1H), 4.63 (bs, 1H), 4.35 (ddd, *J* = 9.3, 7.6, 3.9 Hz, 1H), 4.15–4.03 (m, 3H), 3.89 (dd, *J* = 34.0, 13.5 Hz, 2H), 3.82 (s, 3H), 3.75 (d, *J* = 13.8 Hz, 1H), 3.12–3.00 (m, 1H), 2.90 (dt, *J* = 13.9, 5.3 Hz, 2H), 2.68–2.66 (m, 2H);^13^C NMR (150 MHz, Acetone) δ 149.86, 148.73, 142.36, 140.19, 140.14, 139.53, 128.96(2C), 128.06(2C), 126.76, 125.26, 124.85, 124.15, 122.40, 121.05, 120.70(2C), 118.79, 118.79, 115.10, 113.65, 112.14, 110.39, 109.03, 67.13, 61.38, 59.36(2C), 55.20(2C), 52.65 ppm; HRMS-ESI (m/z): Calculated for C_32_H_34_N_2_O_4_ M^+^: 510.2519, Found: 510.2522.

#### 4-(benzyl(2-(2-methoxyphenoxy)ethyl)amino)-1-(9-methyl-9H-carbazol-4-yl)butane-1,3-diol (**6b**)

^1^H NMR (600 MHz, Acetone) δ 8.54 (d, *J* = 8.0 Hz, 1H), 7.53 (dd, *J* = 10.8, 7.8 Hz, 2H), 7.46 (dt, *J* = 7.9, 4.4 Hz, 2H), 7.43–7.39 (m, 3H), 7.28 (dd, *J* = 10.2, 4.6 Hz, 2H), 7.22 (dd, *J* = 8.3, 6.3 Hz, 1H), 7.18–7.13 (m, 1H), 6.96 (dd, *J* = 7.8, 1.5 Hz, 1H), 6.93–6.88 (m, 2H), 6.87 (dd, *J* = 6.8, 1.3 Hz, 1H), 4.35 (tdd, *J* = 7.8, 5.3, 2.7 Hz, 1H), 4.06 (td, *J* = 5.4, 1.7 Hz, 2H), 3.94–3.88 (m, 4H), 3.82 (s, 3H), 3.74 (d, *J* = 13.7 Hz, 1H), 3.04 (dt, *J* = 12.5, 6.3 Hz, 1H), 2.93–2.86 (m, 2H), 2.67 (dd, *J* = 6.5, 2.6 Hz, 2H), 2.02–1.98 (m, 1H), 1.91–1.88 (ddd, *J* = 14.2, 9.7, 2.5 Hz, 1H); ^13^C NMR (150 MHz, Acetone) δ 149.85, 148.71, 142.47, 141.16, 141.05, 139.46, 128.97 (2C), 128.07 (2C), 126.79, 125.36, 124.96, 124.19, 121.98, 121.06, 120.71, 118.78, 118.47, 115.20, 113.64, 112.14, 108.28, 106.93, 67.68, 67.10, 65.27, 61.35, 59.35, 55.21, 52.65, 42.79, 28.39 ppm; HRMS-ESI (m/z): Calculated for C_33_H_36_N_2_O_4_ M^+^: 524.2675, Found: 524.2677.

#### 1-([1,1′-biphenyl]-3-yl)-4-(benzyl(2-(2-methoxyphenoxy)ethyl)amino)butane-1,3-diol (**6c**)

^1^H NMR (600 MHz, CDCl_3_) δ 7.66–7.58 (m, 3H), 7.52–7.39 (m, 5H), 7.39–7.30 (m, 5H), 7.26 (dd, *J* = 5.0, 3.5 Hz, 1H), 6.97–6.87 (m, 3H), 6.83 (dd, *J* = 7.9, 1.5 Hz, 1H), 5.12 (dd, *J* = 8.0, 3.1 Hz, 1H), 4.09–4.04 (m, 2H), 3.93–3.84 (m, 4H), 3.82 (d, *J* = 11.8 Hz, 1H), 3.71–3.65 (m, 1H), 3.14–3.06 (m, 1H), 2.94 (dt, *J* = 14.1, 4.8 Hz, 1H), 2.69 (dd, *J* = 29.6, 6.7 Hz, 2H), 1.92–1.83 (m, 2H); ^13^C NMR (150 MHz, CDCl_3_) δ 149.48, 148.10, 145.35, 141.24, 141.21, 138.52, 128.94(2C), 128.73, 128.69(2C), 128.40(2C), 127.28, 127.22, 127.20(2C), 125.88, 124.56, 124.47, 121.37, 120.80, 113.23, 111.71, 71.53, 66.91, 65.44, 60.68, 59.56, 55.76, 52.77, 42.34; HRMS-ESI (m/z): Calculated for C_32_H_35_NO_4_ M^+^: 497.2566, Found: 497.2565.

#### Data collection and preparation

Pharmacophore modeling correlates activities with the spatial arrangement of various chemical features in a set of active analogues. Total seven types of 417AChE inhibitors were selected as a training set from ChEMBL database^43^, to generate common features in the pharmacophore models. The *in vitro* bioactivities of the selected inhibitors were expressed as the concentration of the test compounds that inhibited the activity of AChE by 50% (IC_50_). These values are generally transformed into pIC50 (−log IC_50_) as an expression of drug potency. In various assay conditions only datasets that follow Ellman assay method were chosen because it is most common method^58^. Among 82 diverse compounds, 58 were in the range of 0.33–170000 nM were selected as the training set while the remaining 24 molecules served as the test set. The training set molecules play an important role in determining the quality of the pharmacophore models generated; while the test set compounds serve to evaluate the predictive ability of the resultant pharmacophore.

#### Molecular modeling

The Glide SP (version 6.1; Schrödinger software)^59^ protocols used in this study were the procedures described in our laboratory and the methodology for their preparation has been previously studied (unpublished results). We considered acetyl cholinesterase (AChE) and butyrylcholinesterase (BuChE) for this study and check the active site. Acetylcholine (Ach) is found mainly in blood and neuronal synapses, whereas BuChE is found in the liver^60^. AChE had two difference binding sites, a catalytic and a peripheral site, whereas in BuChE there is only the peripheral binding site^61^. On the basis of active site we had classified dataset in to catalytic site, the peripheral site and dual sites^62^. Five crystal structures (PDB ID: 4EY5, 4EY6, 4EY7, 4BDT and 4M0E) (Table 1) were selected and grids created in to same active site where co-ligand bind^63–65^. It was assumed that residue near ligand catalytic binding site is flexible and dependent on ligand structure, showed good docking score. After complying all conformation of 417 dataset entries as potential dual inhibitors, 82 compounds showed best poses with PDB: 4EY7, were selected and considered as bioactive conformers.

#### Molecular docking

The 3D X-ray crystal structures of AChE complex with an inhibitor were retrieved from Protein Data Bank (PDB ID: 4EY7] and prepared using the protein preparation wizard of available in Schrodinger Suite 2015. The protein preparation consisted of fixing structures, deleting unwanted chains and waters, fixing hetero groups and finally optimizing the fixed structure. Hydrogen atoms were added by applying an all atom force field. The force field applied OPLS_2005 and the RMSD of the atom displacement for terminating the minimization was specified as 0.30 Å. The grid was generated for prepared proteins i.e. an all atom structure with appropriate bond orders and formal charges considering the active site residues. The grid-enclosing box was centered on the donepezil and defined to enclose residues located within 10 Ǻ. To test the docking parameters low energy conformations of all compounds were docked into the catalytic pocket of the AChE protein (PDB-ID:4EY7) using Grid-Based Ligand Docking With Energetics (Glide v6.4, Schrödinger 2015-3) in ‘extra precision’ mode without applying any constraints. The final best docked structure was selected using a Glide score function, Glide energy and Glide Emodel energy. Finally, the lowest-energy docked complex of 82/4EY7was selected for further study. Before performing docking studies, Glide XP program was validated by re-docking the co-crystal ligands donepezil with co-crystal protein structure 4EY7. The accuracy of the docking software was measured using RMSD between redocked and co-crystal ligand. The docking poses were selected based on the Glide score.

#### Pharmacophore-based QSAR model

The pharmacophore modelling for AChE was carried out using the PHASE (version 4.3)^66^ module of Schrodinger molecular modelling package. The common pharmacophore hypothesis was identified by dividing the dataset in to active and inactive sets. The ligands with property pIC_50_ were selected as the experimental activity variable in order to build model. The pIC_50_ value was calculated using the formula pIC_50_ = −logIC_50_. The most active and inactive compounds were considered for developing common pharmacophore hypothesis based upon the pIC_50_ values. The maximum and minimum pIC_50_ value was found to be 9.48 and 3.77. The threshold value dividing the total dataset into active and inactive was fixed at a pIC_50_ value of 6.9 by using the kmeans algorithm (Hartigan-Wong method)^67^ in R language (version 3.1.1)^65^. Hence the molecules with pIC_50_ values 6.9 and above were considered active while the molecules with pIC50 values 6.9 and below were considered inactive. Inactive molecules were used for elimination of hypothesis that did not provide good explanation of activity on the basis of pharmacophore alone. Active set determined the pool of pharmacophore models generated and the initial scores assigned to them.

The pharmacophore features in the ligand conformations used for hypothesis generation included Hydrogen bond acceptor (A), Hydrogen bond donor (D), Hydrophobic group (H), Positively ionisable (P), Negatively ionisable (N) and aromatic rings (R) defined by a set of chemical structure patterns. The pharmacophore of active ligands that contain identical sets of features with very similar spatial arrangements were grouped together to give rise to a common pharmacophore hypothesis (CPH). A common 6-point or 5 site pharmacophore with a terminal box size of 1 Å was considered. The most active ligand was taken as the reference ligand showing highest activity and fitness score 1. The inactive/non-modelled molecules in the dataset were aligned, based on the matching of at least six of the pharmacophore features. The maximum and minimum numbers of sites were set to be 5 and 3 respectively. A common pharmacophore is matched to a subset of active ligands when the actives are highly diverse. The common pharmacophore matched a minimum required number of actives which was set to be 55 out of 82 active ligands. The feature frequencies table denoted the number of times a feature allowed to appear in a common pharmacophore. The minimum and maximum limit was set to 0 and 5 respectively. The list of variants denoted the possible combinations of features that could give rise to common pharmacophore; about 21 variants were selected. A common pharmacophore model AAHPRR.15 for AChE was generated after the creation and identification of pharmacophoric sites in all the molecules of the dataset.

#### Model validation

In general, pharmacophore models should be statistically significant, accurately predict the activity of molecules and retrieve active compounds from databases. The best pharmacophore model was validated using various potent approaches such as Fischer’s randomization, decoy set^68^ and Güner-Henry (GH) scoring method^32,69,70^. The main purpose of validating a quantitative pharmacophore model is to determine its capacity to identify active compounds, as well as its predictive ability for corresponding molecules. Fischer’s randomization test was performed simultaneously during the original hypotheses generation and produced a number of random spreadsheets depending on the selected significance level (90%, 95%, 98% and 99%) by shuffling the activity values present in the training set. In order to obtain the multi-dimensional descriptor, Sybyl-X (Tripos, St Louis, MO, US) was used^71^. A Sarmap graph generated using unity fingerprint based on pIC_50_and highest value found red color. All compounds randomly divided in to seven groups based on sarmap descriptors^70^. After statistical regression analysis selected best pharamcophore model based on highest R-square and Q-square value^72^.

The QSAR model AAHPRR. 15 with 6 components PLS factor was characterized as the best model (Table 2). The pharmacophoric model was validated by its accuracy in predicting the training set ligands activity (Table 1). The predicted AChE inhibitor activity of training set ligands exhibited a correlation (R^2^) of 0.73 with observed AChE inhibitor activity. Scatter plots for the experimental and predicted activities of ligands exhibited satisfactory linear correlation and moderate difference between experimental and predicted values (Supplementary Table 2). The efficacy of model AAHPRR.15 was further examined with the external validation (Fig. 3A,B). The generated 3-point model predictive power was further tested against 1000 decoy test set compounds^73^ retrieved from the ZINC data base^74^. Enrichment factor (EF) and Robust initial enhancement (RIE) were calculated to benchmark the reliability of the model and for the accurate ranking of compounds^75^ (Table 3 and Fig. 4B).

#### Virtual screening

The selected pharmacophore hypothesis was used to search Drug-like diverse database comprising 417 molecules in the Phase (version 4.3; Schrödinger software)^62,66^. We had chosen ‘best’ search method to select pre-generated diverse conformations using OPLS_2005 force field and energy tolerance of 20 kcal/mol. The alignment of hypothesis features with database molecules was enabled to prevent the recognition of molecules only with the mere presence of features as hits, with the minimum inter-feature distance of 2.0 Å. This screening protocol generated a list of hit molecules sorted by fit value among which the top fourteen hits were selected for further study. The validated pharmacophore-AChE models were used as 3D queries in an in-house chemical database screening to retrieve potential selective inhibitors. In-house chemical contained six compounds that had been synthesized by our team including four serials. The compounds were filtered by Lipinski’s “Rule of five” that sets the criteria for drug-like properties. Drug likeness is a property that is most often used to characterize novel lead compounds by screening of structural libraries^34,36,37^. According to this rule, poor absorption is expected if MW > 500, log P > 5, hydrogen bond donors >5 and hydrogen bond acceptors >10. Phase v4.3 of complex based pharmacophore was used to screen the database consisting of the remaining 417 compounds filtered by ADMET properties^38–40^. The number of conformations was set to 200, while the conformation method was set to BEST. The minimum interfeature distance was set to 2. The number of limit hits was set to first N and the maximum number of hits was set to 500. Thus, the top 58 (pharmacophore) molecules were used to compare with docking simulations. In the first step, the high-throughput virtual screening mode of Glide was used and the remaining 10% of the top-scoring ligands were further subjected to Glide SP docking. Again, 10% of the top-scoring leads from Glide SP were retained and all the ligands were subjected further to Glide XP docking. Only hits with a docking score less than −5.2 were retained. During the docking process, the docking score was used to select the best conformation for each ligand. The accuracy of the model was validated by the Güner-Henry scoring method using known actives and a decoy set.

Selected compounds had at least three features that matched the model. Compounds with vector scores under −1.0 or volume scores under 0 were rejected. Vector score (Svec) was calculated by mean of the cosine of the angles of each pharmacophore feature, such as acceptors, donors, rings, compared to the model. Volume score (Svol) calculated how much each pharmacophore’s features overlapped with the model’s and search mean of each volume scores. The align score was a RMSD in the site point positions; Svec represents vector score and averages the cosine of the angles formed by corresponding pairs of vector features in aligned structures; Svol represents volume score based on overlap of van der Waals models of non-hydrogen atoms in each pair of structures and accounts for what fractions of molecules are likely to match the hypothesis regardless of their activity towards the receptorin the matching conformation and the site point positions in the hypothesis. Fitness score was computed using the align score, vector score and volume score. The equation 1 is defined by:

1 $${\rm{S}}={W}_{site}\ast (1-\frac{{S}_{align}}{{C}_{align}})+{W}_{vec}\ast {S}_{vec}+{W}_{vol}\ast {S}_{vol}$$

W_site_ = weight of each feature point. If some feature is important and powerful, it should be above 1. Generally, it is set as one and this study concurred. The cut off alignment (C_align_) score was set by default to 1.2. All weight factors were set by default to 1. If vector score was under −1.0 or the volume score was under zero, the result would be rejected by default.

#### ADME Prediction

QikProp version 4.6 (Maestro)^76^ computes unique ADME (Adsorption, Distribution, Metabolism and Excretion) relevant descriptors^34,37,38^. It may prove to be a novel tool in optimizing the pharmacokinetic profile for pharmaceutically relevant compounds. The significance of favorable pharmacokinetic features for the discovery of a successful drug has been widely acknowledged in the last few years, such that ADME assessments are integrated earlier into drug discovery and design strategies. We assessed the ADME properties of active compounds by using QikProp(version 4.7; Schrödinger software)^36^. The QikProp program utilizes *in vitro* results and its classification schemes related to Human Intestinal Absorption [HIA (%)], MDCK cell model [Madin-Darby Canine Kidney cell (nm/sec)], cell permeability from Caco-2 [human colon adenocarcinoma cells possessing multiple drug transport pathways through the intestinal epithelium (nm/sec)] and distribution for Plasma Protein Binding [PPB (%)] and Blood-Brain Barrier Inhibition of AChE penetration [BBB; C.brain/C.blood] prediction.

### Acetylcholinesterase and butyrylcholinesterase inhibition assay

Acetylcholinesterase from electrophorus electricus (electric eel), horse butyrylcholinesterase, Acetylcholine iodide, S-butyrylthiocholine chloride and 5,5′-dithio-bis-nitrobenzoic acid (DTNB) were purchased from the Sigma (St. Louis, MO) while bovine serum albumin (BSA) purchased from Research and Diagnostic Technology (RDT). Buffers and other chemicals were purchases from pure chemicals as a pure experimental grade. All the reagents and experimental conditions were same as described by previous reports^77^. Tacrine was used as standard compound. Acetylcholinesterase and butyrylcholinesterase inhibition was performed spectrometrically using acetylthiocholine and butyrylthiocholine as a substrate. Experiment was performed with slight modification in the method described by Ellman *et al*.^78^. Then, 0.1 mM sodium phosphate buffer (pH 8.0), enzyme preparation and different concentration of test compound was mixed and incubated for 30 min followed by the addition of DTNB and the reactions was initiated by adding respective enzyme substrate like acetylthiocholine iodide, butyrylthiocholine chloride for Acetylcholinesterase and butyrylcholinesterase inhibition assay respectively. The hydrolysis of acetylthiocholine or butyrylthiocholineyield a dark yellow 5-thio-2-nitrobenzoate anion because of the reaction between DTNB with enzyme and enzyme substrate. It was quantified at a wavelength of 412 nm. Solvent used for compound dilution was used for the negative control. Percentage inhibition was calculated taking negative control as 100%. Tacrine was used as positive control for Acetylcholinesterase and butyrylcholinesterase inhibition assay^54,79^.

## Electronic supplementary material

Supplementary Data
