# Supplementary material for: Identification of novel acetylcholinesterase inhibitors designed by pharmacophore-based virtual screening, molecular docking and bioassay
Source: Sci Rep. 2018 Oct 8;8:14921. doi: 10.1038/s41598-018-33354-6 (PMC6175823; doi:10.1038/s41598-018-33354-6)
Supplement: Supplementary file 1 — Supplementary Data [file 41598_2018_33354_MOESM1_ESM.pdf]

Identification of novel acetylcholinesterase inhibitors designed by pharmacophore-based virtual screening, molecular docking and bioassay

Cheongyun Jang, Dharmendra K. Yadav, Lalita Subedi, RamuVenkatesan, Arramshetti Venkanna, Sualiha Afzal, Eunhee Lee, JaewookYoo, Eunhee Ji, Sun Yeou Kim, Mi-hyun Kim*

Gachon Institute of Pharmaceutical Science and Department of Pharmacy, College of Pharmacy, Gachon University, Yeonsu-gu, Incheon, Republic of Korea

* Author for correspondence

E-mail: kmh0515@gachon.ac.kr

S.No	Contents	Page No
1	Supplementary table	3
2	Supplementary table 2	5
3	Supplementary Figure 1	12
4	Supplementary Figure 2	12
5	Supplementary Figure 3	13
6	Supplementary Figure 4	13
7	Supplementary Figure 5	14
8	Supplementary Figure 6	15
9	Supplementary Figure 7	15
9	NMR Spectra	16
10	Mass Spectra	24

Supplementary table

Table 1: Pharmacophore features, intersite distances and angles of AAHPRR.15 model

Site1	Site2	Distance	Site1	Site2	Site3	Angle	Site1	Site2	Site3	Angle	Site1	Site2	Site3	Angle
A2	A3	4.603	A3	A2	H5	44.1	A2	H5	A3	94.3	A2	R7	A3	75.1
A2	H5	3.068	A3	A2	P6	95	A2	H5	P6	100	A2	R7	H5	36.8
A2	P6	6.266	A3	A2	R7	52.6	A2	H5	R7	47.4	A2	R7	P6	23
A2	R7	3.768	A3	A2	R8	94.1	A2	H5	R8	109.1	A2	R7	R8	23.6
A2	R8	8.979	H5	A2	P6	51.1	A3	H5	P6	164.4	A3	R7	H5	39
A3	H5	3.212	H5	A2	R7	95.8	A3	H5	R7	47.9	A3	R7	P6	56.2
A3	P6	8.094	H5	A2	R8	52	A3	H5	R8	149.1	A3	R7	R8	51.5
A3	R7	3.785	P6	A2	R7	143.5	P6	H5	R7	144.2	H5	R7	P6	17.7
A3	R8	10.381	P6	A2	R8	20.8	P6	H5	R8	27.3	H5	R7	R8	14.8
H5	P6	4.954	R7	A2	R8	146.7	R7	H5	R8	155.2	P6	R7	R8	14.2
H5	R7	5.094	A2	A3	H5	41.7	A2	P6	A3	34.5	A2	R8	A3	26.2
H5	R8	7.493	A2	A3	P6	50.5	A2	P6	H5	28.8	A2	R8	H5	18.8
P6	R7	9.561	A2	A3	R7	52.3	A2	P6	R7	13.6	A2	R8	P6	35.5
P6	R8	3.835	A2	A3	R8	59.6	A2	P6	R8	123.7	A2	R8	R7	9.7

R7	R8	12.304	H5	A3	P6	9.5	A3	P6	H5	6.1	A3	R8	H5	9.1
			H5	A3	R7	93.1	A3	P6	R7	22.9	A3	R8	P6	44.3
			H5	A3	R8	21.7	A3	P6	R8	116.3	A3	R8	R7	16.6
			P6	A3	R7	100.9	H5	P6	R7	18.2	H5	R8	P6	36.3
			P6	A3	R8	19.3	H5	P6	R8	116.4	H5	R8	R7	10
			R7	A3	R8	111.9	R7	P6	R8	128.1	P6	R8	R7	37.7

Table 2: Calculated pIC_{50} for compounds in the external test sets

Structure	Name	Exp. pIC50	Pred. pIC50	Residual activity	QSAR Set	Pharm Set	Fitness
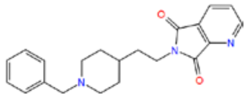	CHEMBL140106	7.886	7.45	0.436	test		2.42
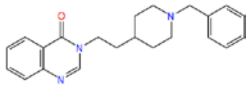	CHEMBL140328	5.921	6.69	0.769	training	inactive	1.93
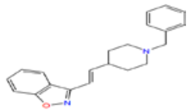	CHEMBL126939	6.678	6.89	0.212	training	inactive	1.9
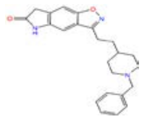	CHEMBL328468	9.022	8.19	0.832	test		1.96
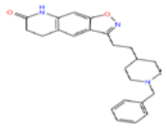	CHEMBL329231	9.244	8.88	0.364	training	active	1.95
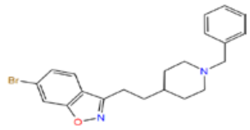	CHEMBL330004	7.301	7.87	0.569	training	active	2.01
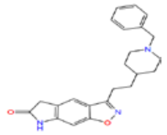	CHEMBL359570	9.481	8.59	0.891	test		1.95
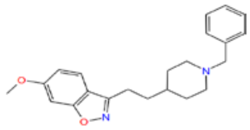	CHEMBL418955	8.056	8.02	0.036	training	active	2.02
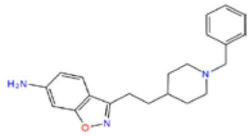	CHEMBL420625	7.699	8.08	0.381	test		2.02
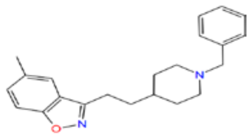	CHEMBL433379	8.108	7.93	0.178	training	active	2.01
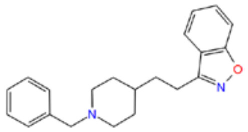	CHEMBL92460	7.26	7.67	0.41	training	active	2

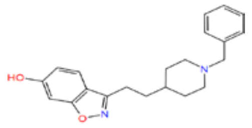	CHEMBL92463	7.585	7.67	0.085	training	active	2.02
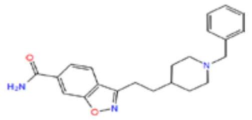	CHEMBL92663	8.056	6.85	1.206	training	active	1.98
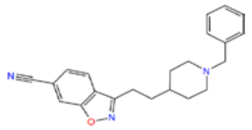	CHEMBL92775	6.996	8.06	1.064	test		2.01
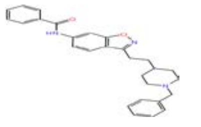	CHEMBL93123	8.027	8.92	0.893	training	active	1.82
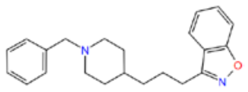	CHEMBL93241	6.046	5.52	0.526	training	inactive	1.8
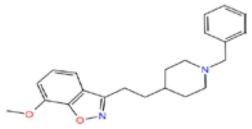	CHEMBL93619	8.149	7.48	0.669	test		2.01
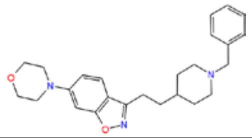	CHEMBL93936	9.097	8.57	0.527	training	active	1.94
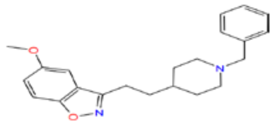	CHEMBL94186	8.143	7.76	0.383	test		2
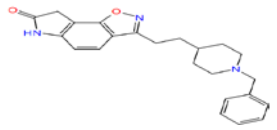	CHEMBL95020	8.444	8.21	0.234	training	active	2.05
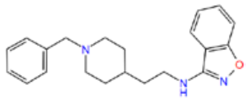	CHEMBL328715	6.092	5.89	0.202	test		1.83
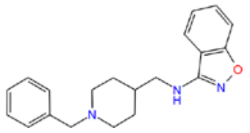	CHEMBL92629	6.495	6.7	0.205	training	inactive	1.92
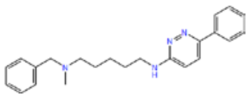	CHEMBL1083057	6.131	6.33	0.199	training	inactive	1.62

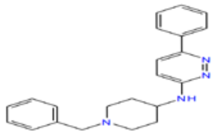	CHEMBL1083058	4.187	5.03	0.843	test		1.61
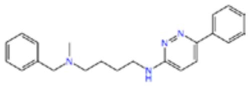	CHEMBL1083367	4.252	4.19	0.062	training	inactive	1.71
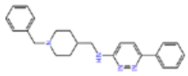	CHEMBL1084257	4.959	5.6	0.641	test		1.69
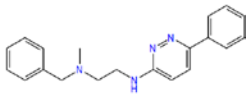	CHEMBL1086185	3.77	4.36	0.59	training	inactive	1.7
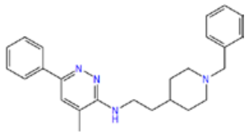	CHEMBL94054	6.495	6.91	0.415	training	inactive	1.77
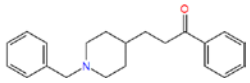	CHEMBL1082433	6.516	7	0.484	training	inactive	1.89
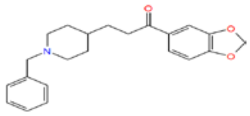	CHEMBL1082434	7.523	7.71	0.187	training	active	1.87
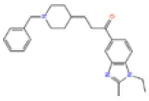	CHEMBL1082435	8.367	7.56	0.807	test		1.73
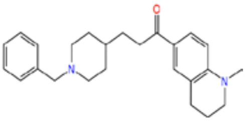	CHEMBL1083660	7.62	7.94	0.32	training	active	1.83
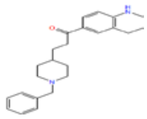	CHEMBL1083661	7.268	7.54	0.272	training	active	1.78
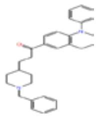	CHEMBL1083662	7.268	7.9	0.632	training	active	1.97
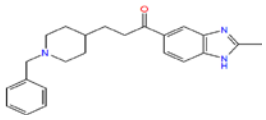	CHEMBL1084790	7.921	7.96	0.039	training	active	1.9

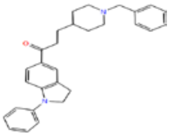	CHEMBL1085858	7.167	7.02	0.147	training	active	1.86
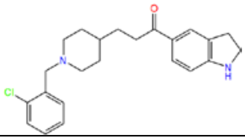	CHEMBL306175	7.119	7.82	0.701	test		1.82
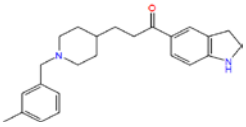	CHEMBL306179	7.301	7.54	0.239	training	active	1.73
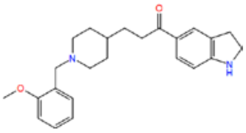	CHEMBL306776	6.09	6.83	0.74	training	inactive	1.86
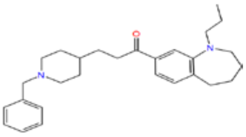	CHEMBL307513	5.876	6.71	0.834	test		1.77
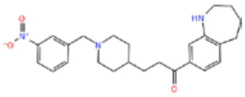	CHEMBL307832	6.409	6.6	0.191	training	inactive	1.9
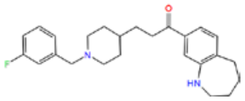	CHEMBL308093	6.839	7.18	0.341	training	inactive	1.76
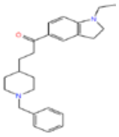	CHEMBL308793	7.959	8.22	0.261	training	active	1.8
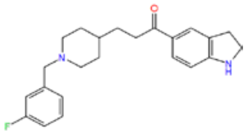	CHEMBL308807	7.796	7.89	0.094	test		1.81
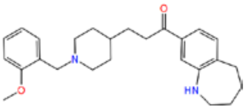	CHEMBL309173	5.576	6.73	1.154	training	inactive	1.72
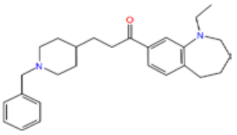	CHEMBL310467	6.425	6.77	0.345	training	inactive	1.82
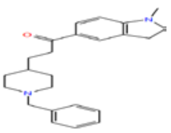	CHEMBL311017	8	8.08	0.08	training	active	1.81

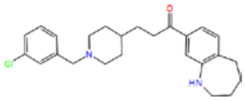	CHEMBL420378	6.478	7.27	0.792	training	inactive	1.79
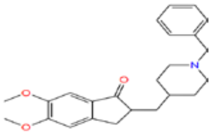	CHEMBL502	8.097	7.78	0.317	training	active	1.98
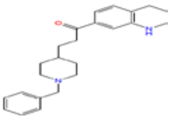	CHEMBL73593	6.785	7.56	0.775	test		1.81
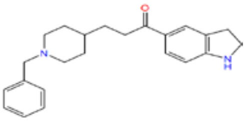	CHEMBL75110	7.699	7.71	0.011	test		1.89
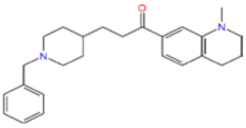	CHEMBL76470	7.444	7.35	0.094	training	active	1.84
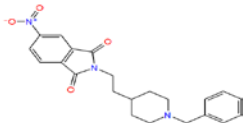	CHEMBL105874	7.903	7.78	0.123	test		2.39
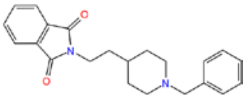	CHEMBL106891	7.097	7.56	0.463	training	active	2.39
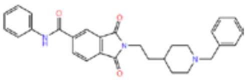	CHEMBL1084256	8.658	8.95	0.292	training	active	2.18
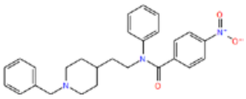	CHEMBL126354	8.268	6.63	1.638	training	active	1.76
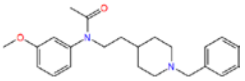	CHEMBL127527	7.337	6.58	0.757	test		1.71
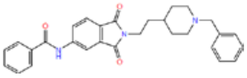	CHEMBL138107	8.921	8.12	0.801	training	active	3
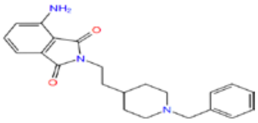	CHEMBL138442	7.959	7.64	0.319	test		2.4

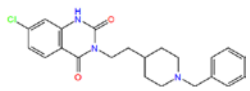	CHEMBL138552	8.347	7.61	0.737	training	active	2.35
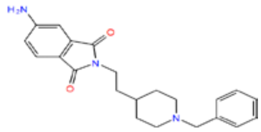	CHEMBL139353	8.056	7.83	0.226	training	active	2.42
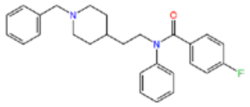	CHEMBL140737	7.745	6.6	1.145	training	active	1.63
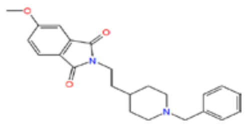	CHEMBL140770	8.097	8.38	0.283	test		2.38
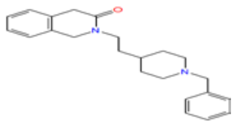	CHEMBL140990	7.77	7.52	0.25	training	active	1.93
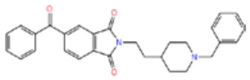	CHEMBL140999	8.62	7.96	0.66	training	active	2.09
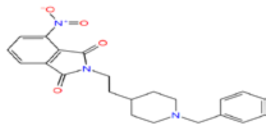	CHEMBL141001	8.046	6.83	1.216	test		2.47
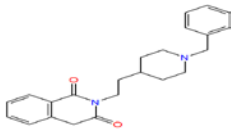	CHEMBL141042	7.638	7.37	0.268	training	active	2.32
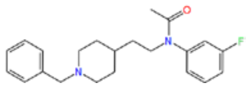	CHEMBL141167	7.187	6.48	0.707	training	active	1.69
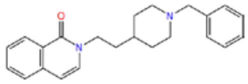	CHEMBL141203	5.959	6.79	0.831	test		1.96
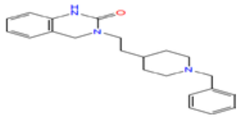	CHEMBL141276	7.886	7.19	0.696	training	active	1.95
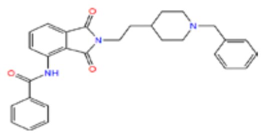	CHEMBL141622	6.469	6.32	0.149	training	inactive	2.05

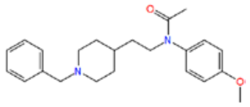	CHEMBL141801	6.155	7.29	1.135	test		1.71
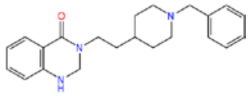	CHEMBL141810	6.097	6.58	0.483	training	inactive	2.24
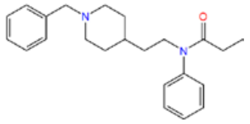	CHEMBL141853	6.081	6.53	0.449	test		1.52
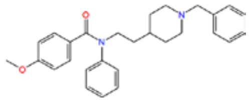	CHEMBL141873	6.229	6.38	0.151	training	inactive	1.64
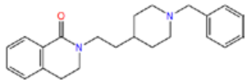	CHEMBL142014	6	7.07	1.07	test		2.11
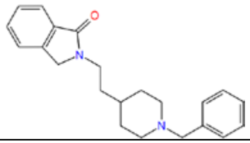	CHEMBL333909	7.009	7.35	0.341	training	active	2.17
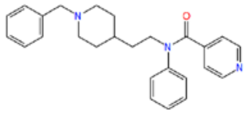	CHEMBL340621	7.194	6.63	0.564	training	active	1.65
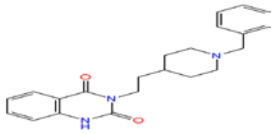	CHEMBL342413	8.377	7.95	0.427	test		2.2
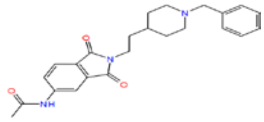	CHEMBL433678	8.553	7.99	0.563	training	active	2.33
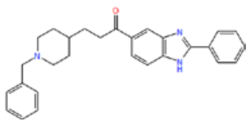	CHEMBL1082436	7.481	7.38	0.101	test		1.69
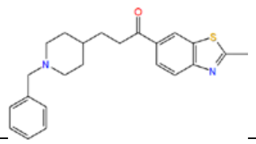	CHEMBL1084275	8.167	8.1	0.067	training	active	1.86

Supplementary Figure :

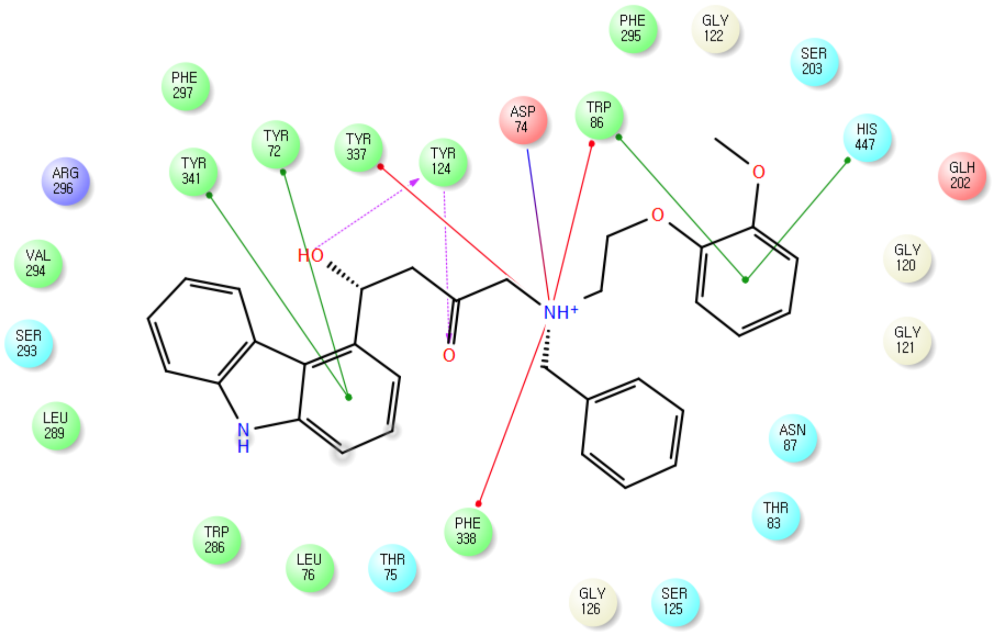

Figure SF 1: 2D-ligand interaction diagram of compound **5a** in the catalytic and peripheral pocket of 4EY7

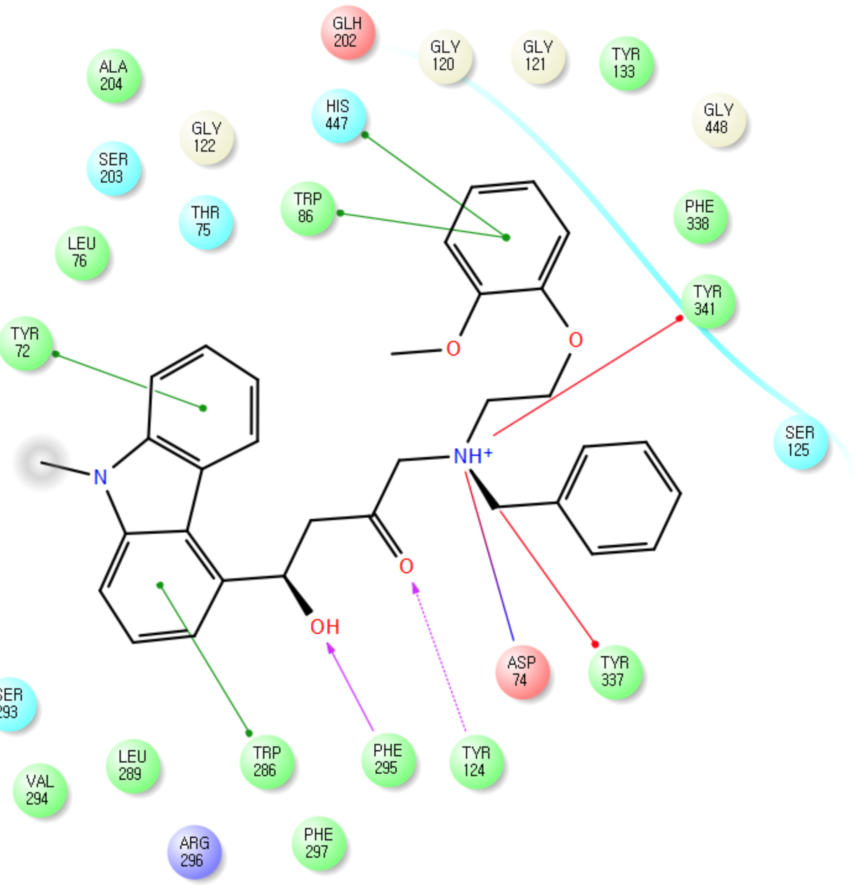

Figure SF 2: 2D-ligand interaction diagram of compound **5b** in the catalytic and peripheral pocket of 4EY7

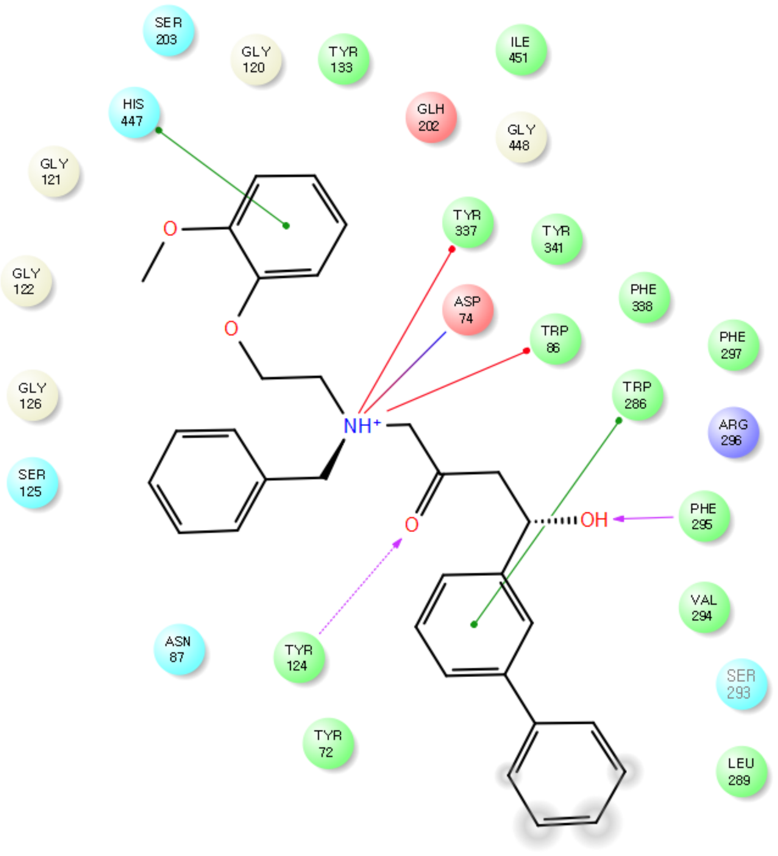

Figure SF 3: 2D-ligand interaction diagram of compound **5c** in the catalytic and peripheral pocket of 4EY7

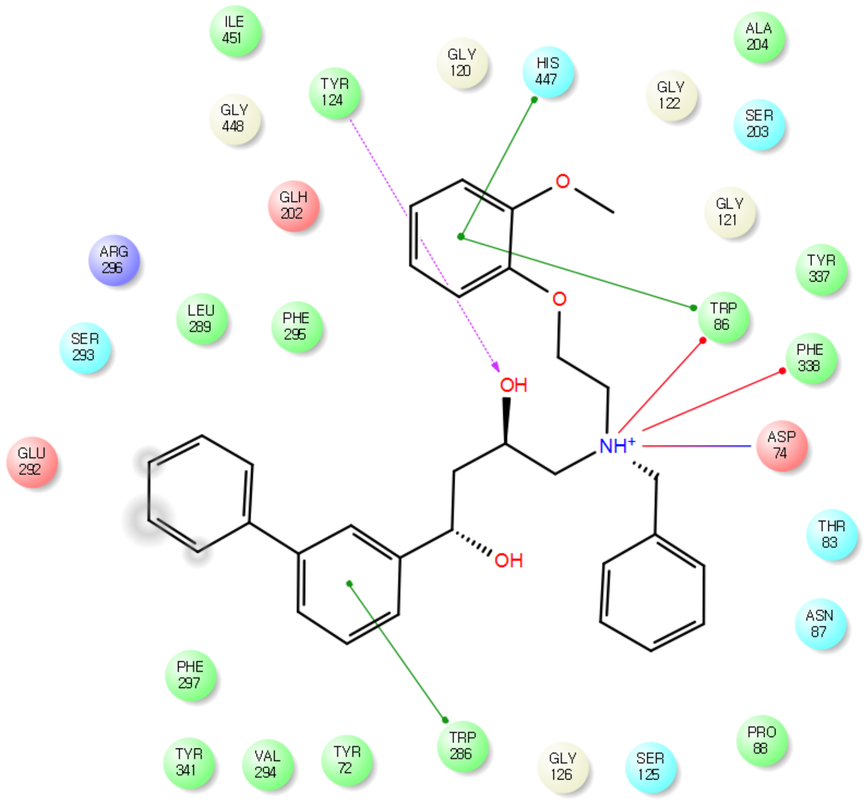

Figure SF 4: 2D-ligand interaction diagram of compound 6**c** in the catalytic and peripheral pocket of 4EY7

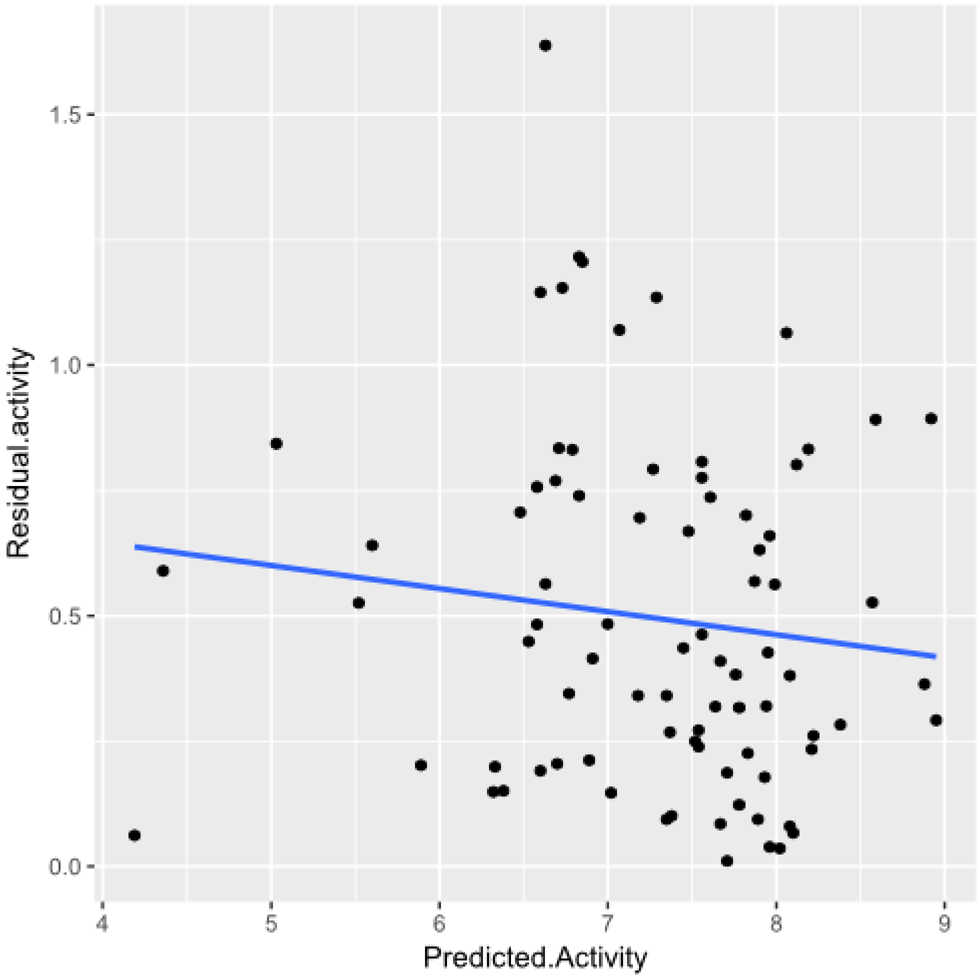

Figure SF 5: Plot of residual value *vs.* predicted value.

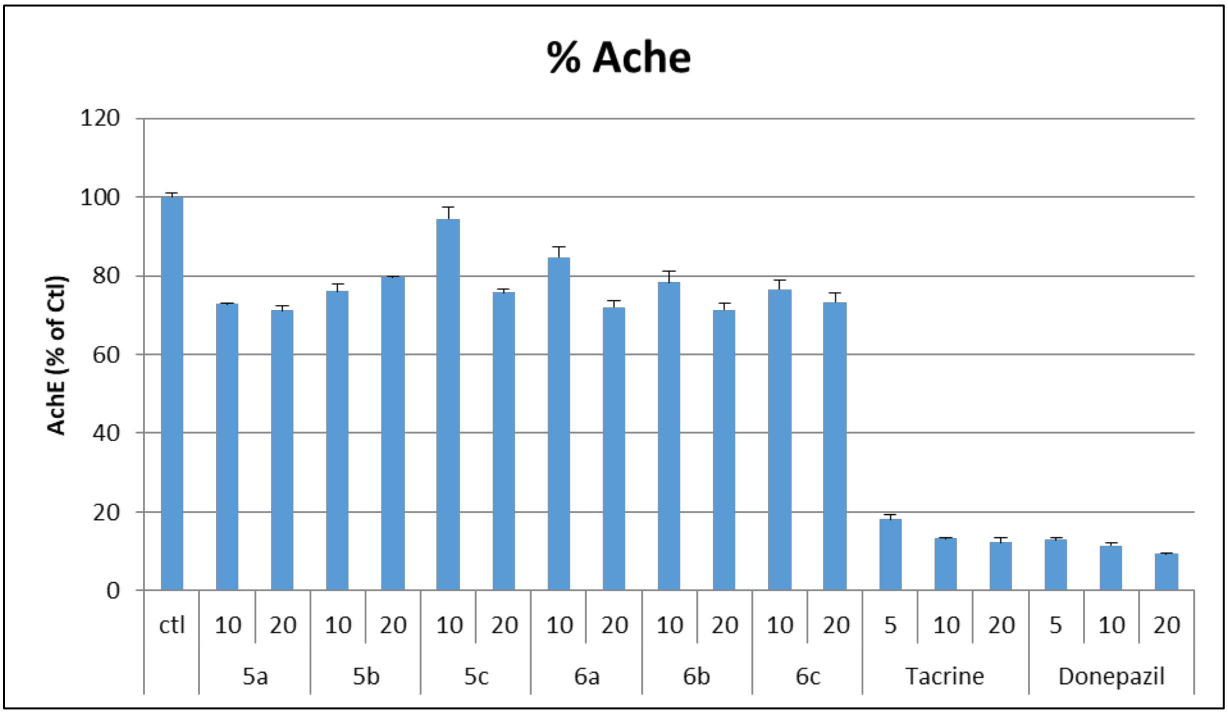

Figure SF 6: AChE % inhibitory activity profile of tested compounds at 10 µM and 20 µM (n = 2).

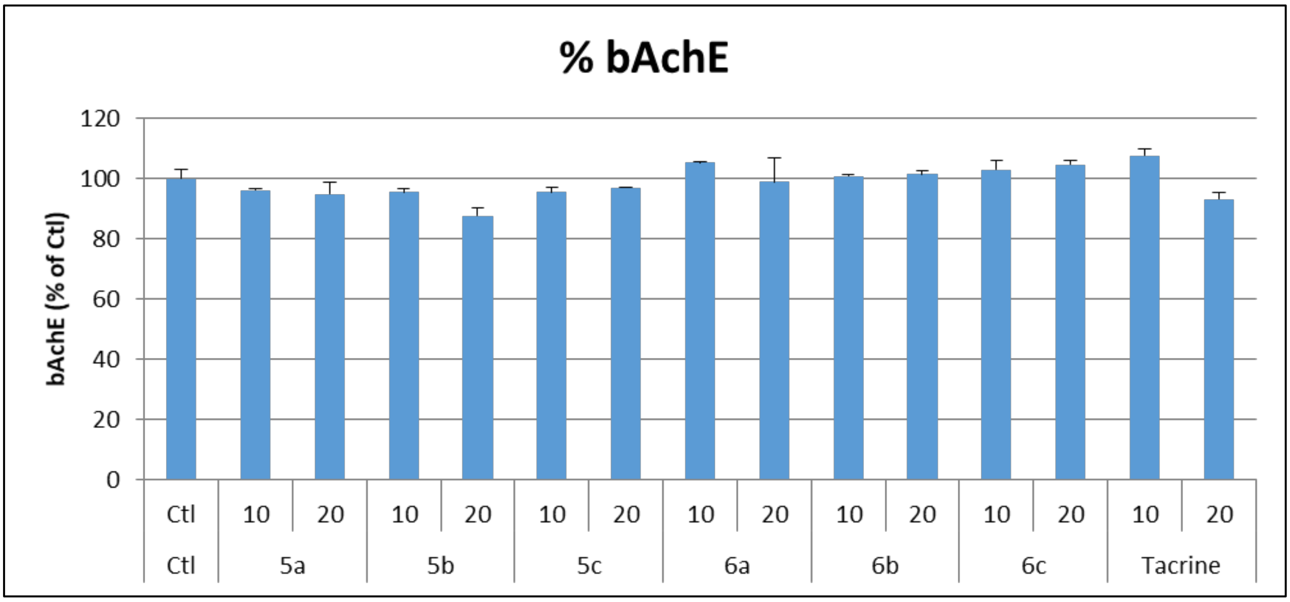

Figure SF 7: bAChE % inhibitory activity profile of tested compounds at 10 µM and 20 µM (n = 2).

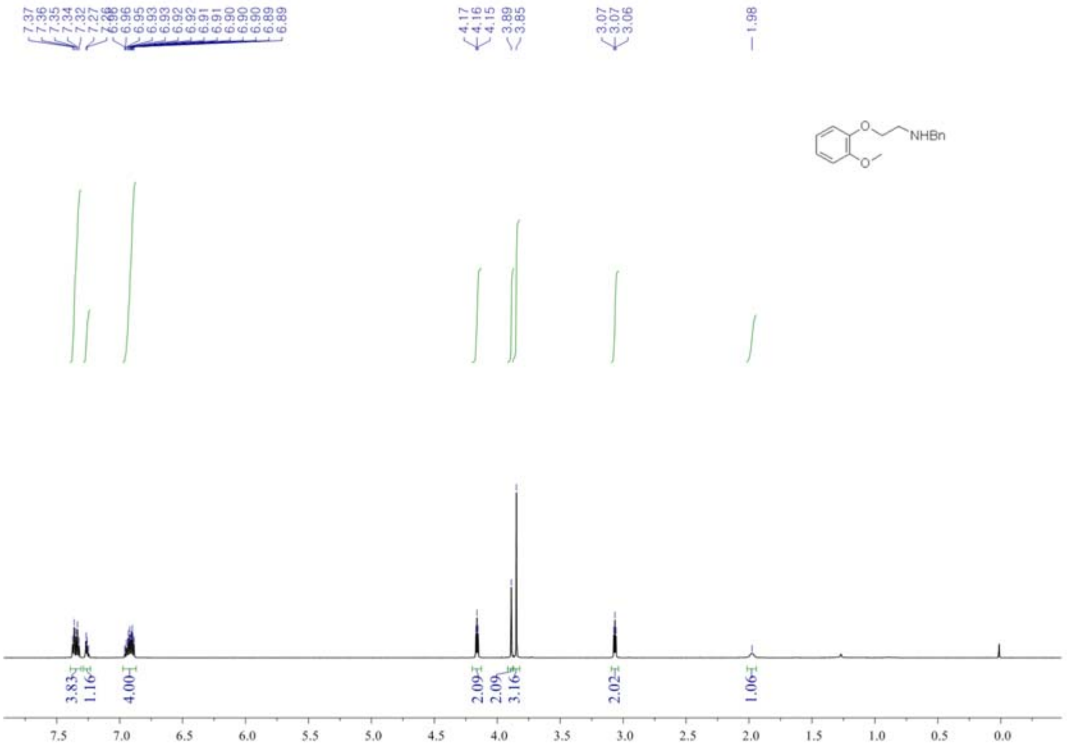

^{1}H NMR Spectrum of compound 2 in $CDCl_3$

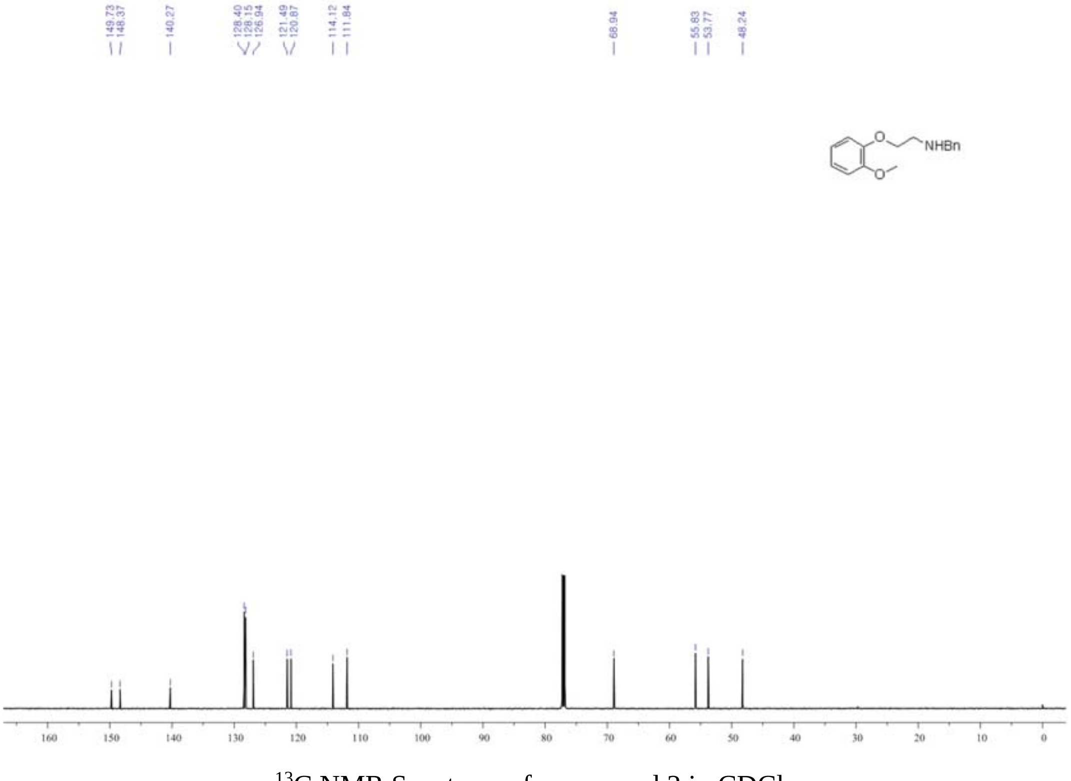

^{13}C NMR Spectrum of compound 2 in $CDCl_3$

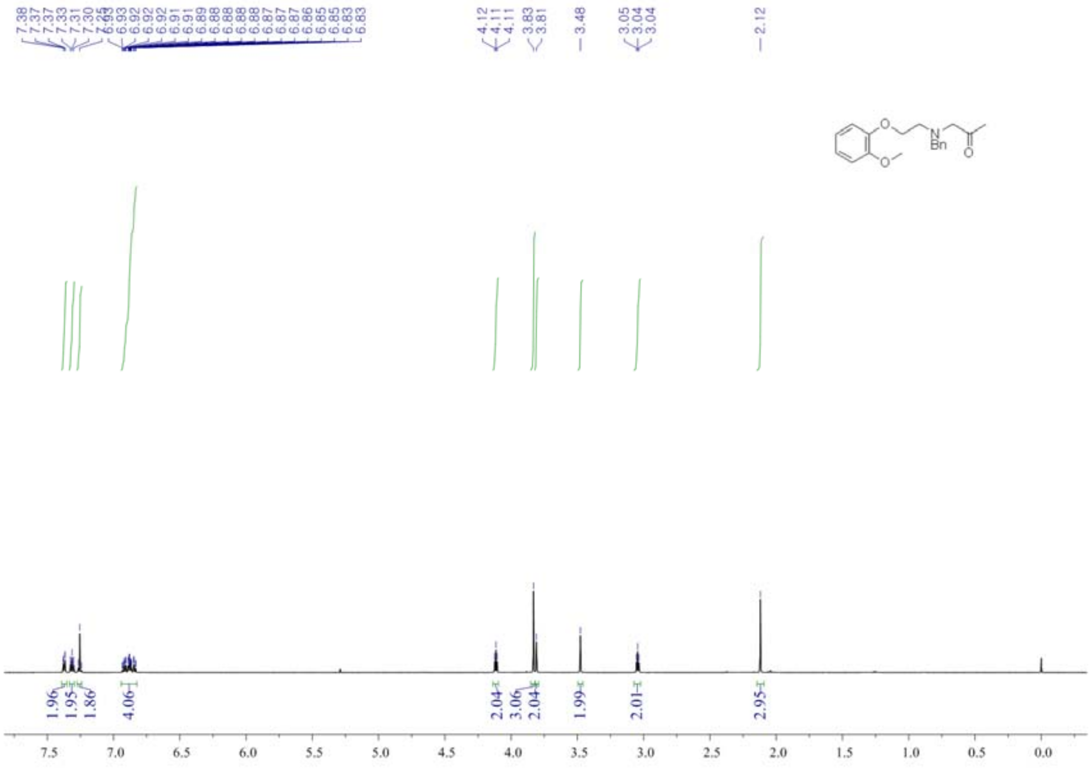

^{1}H NMR Spectrum of compound 3 in $CDCl_3$

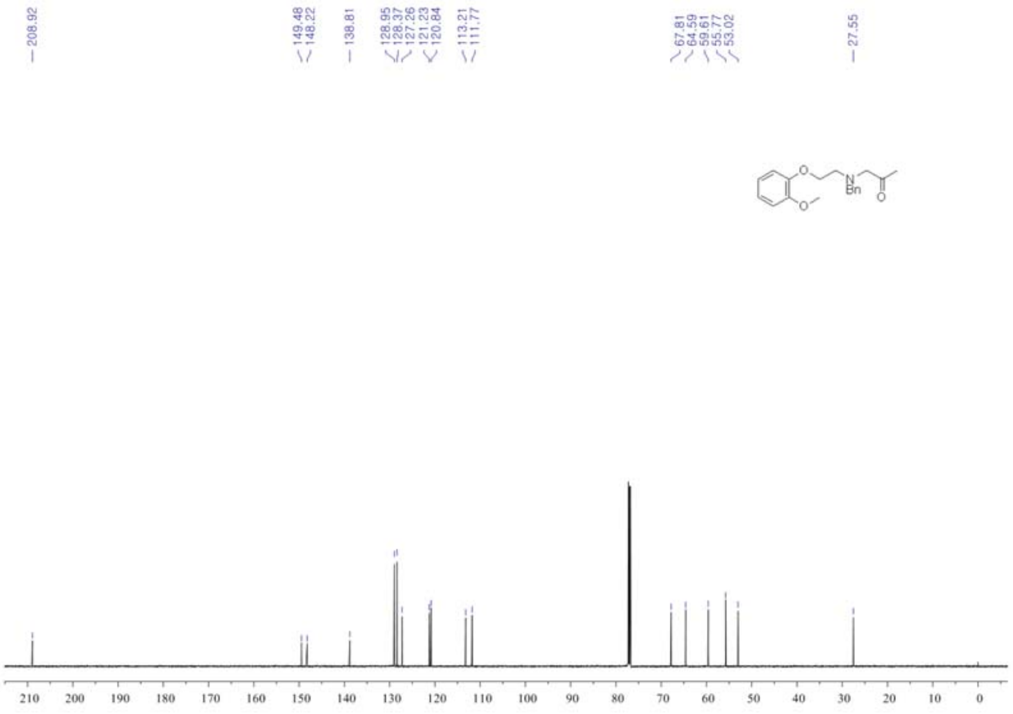

^{13}C NMR Spectrum of compound 2 in $CDCl_3$

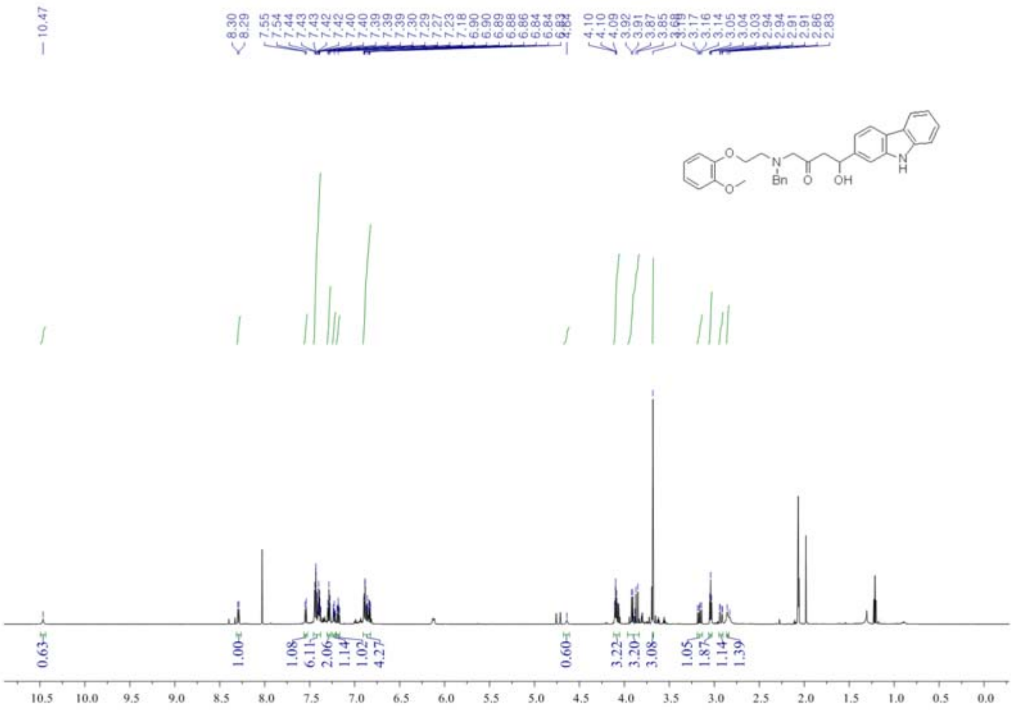

^{1}H NMR Spectrum of compound 5a in Acetoned6

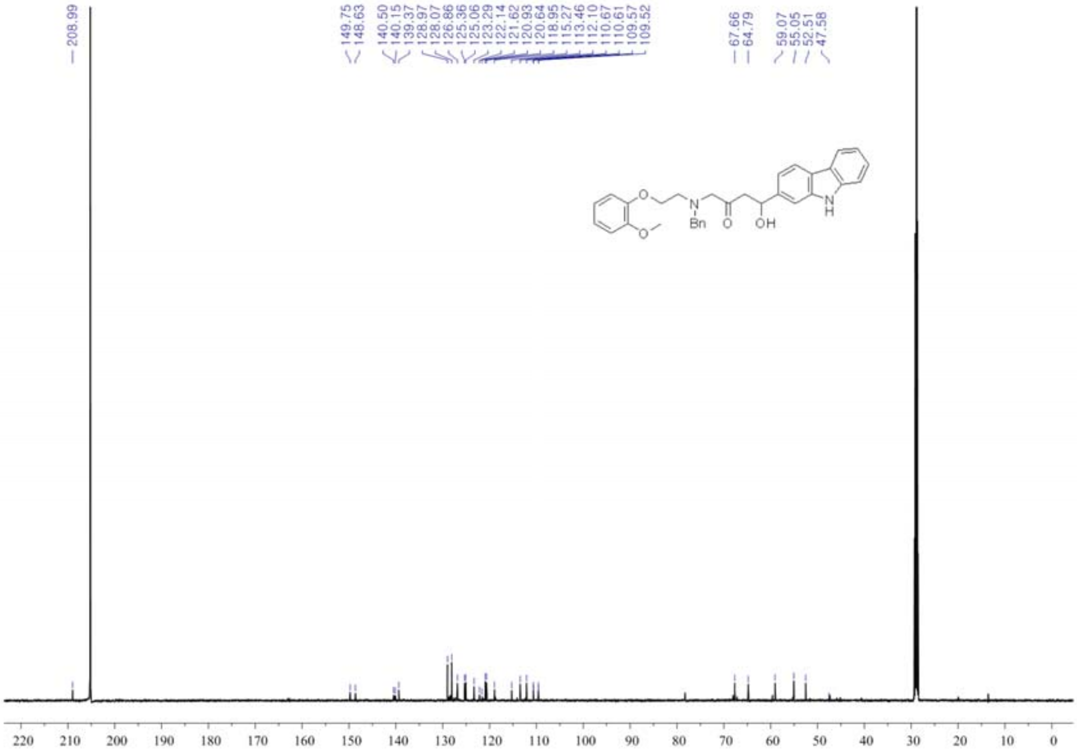

^{13}C NMR Spectrum of compound 5a in Acetoned6

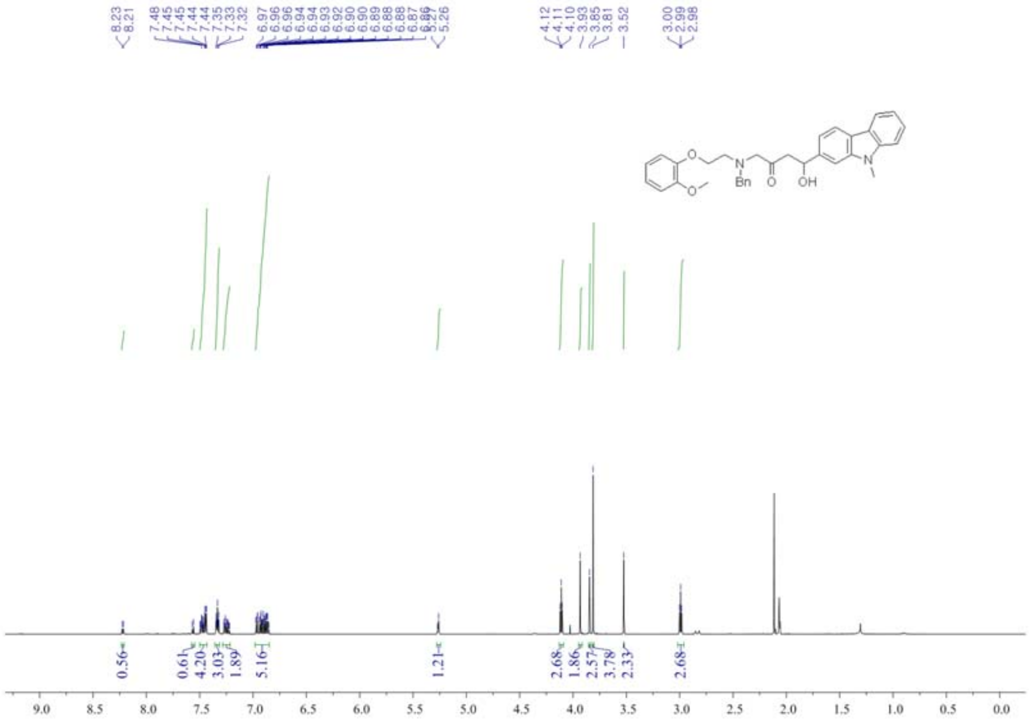

^{1}H NMR Spectrum of compound 5b in Acetoned6

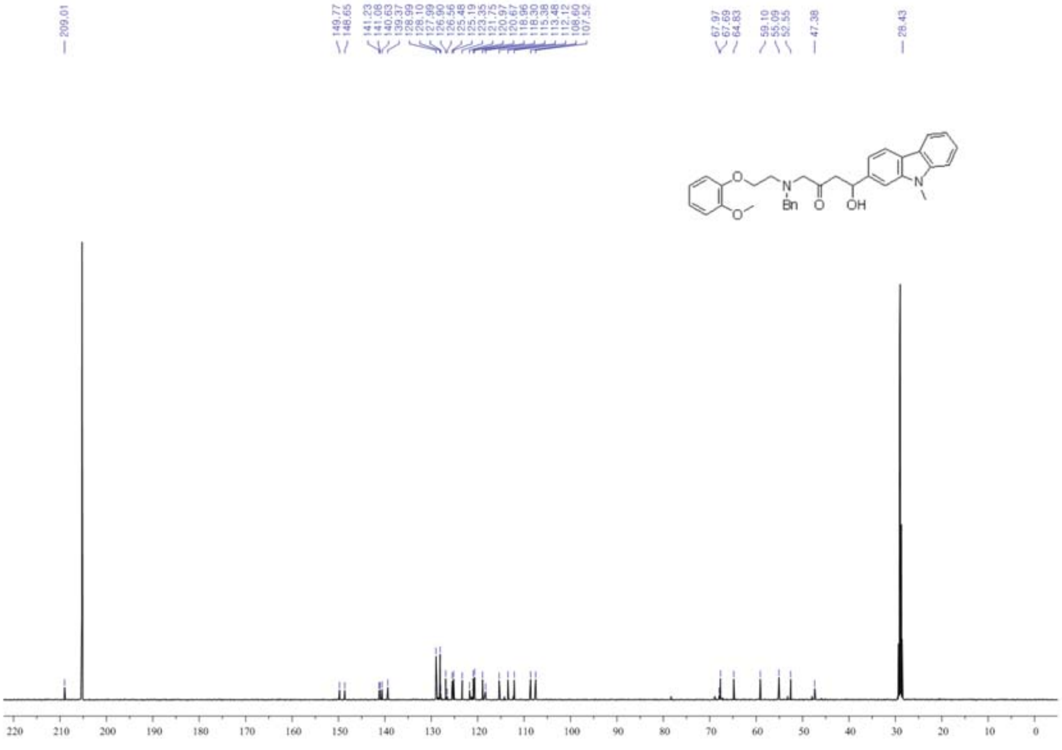

^{13}C NMR Spectrum of compound 5a in Acetoned6

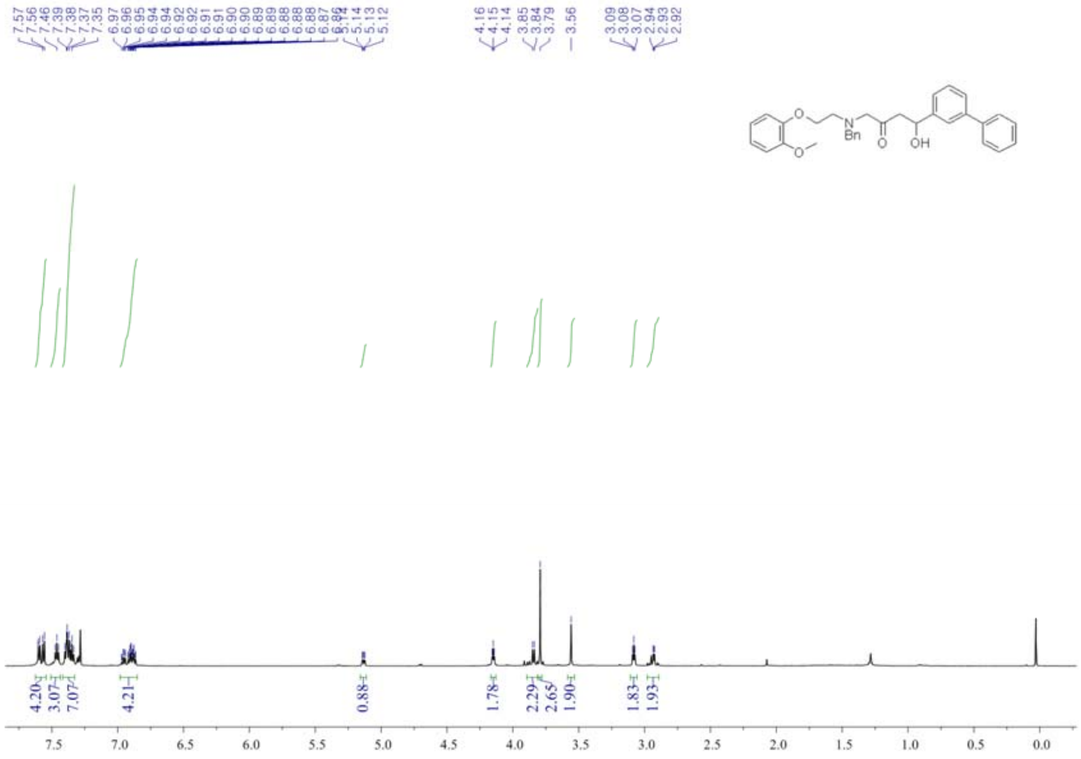

^{1}H NMR Spectrum of compound 5c in $CDCl_3$

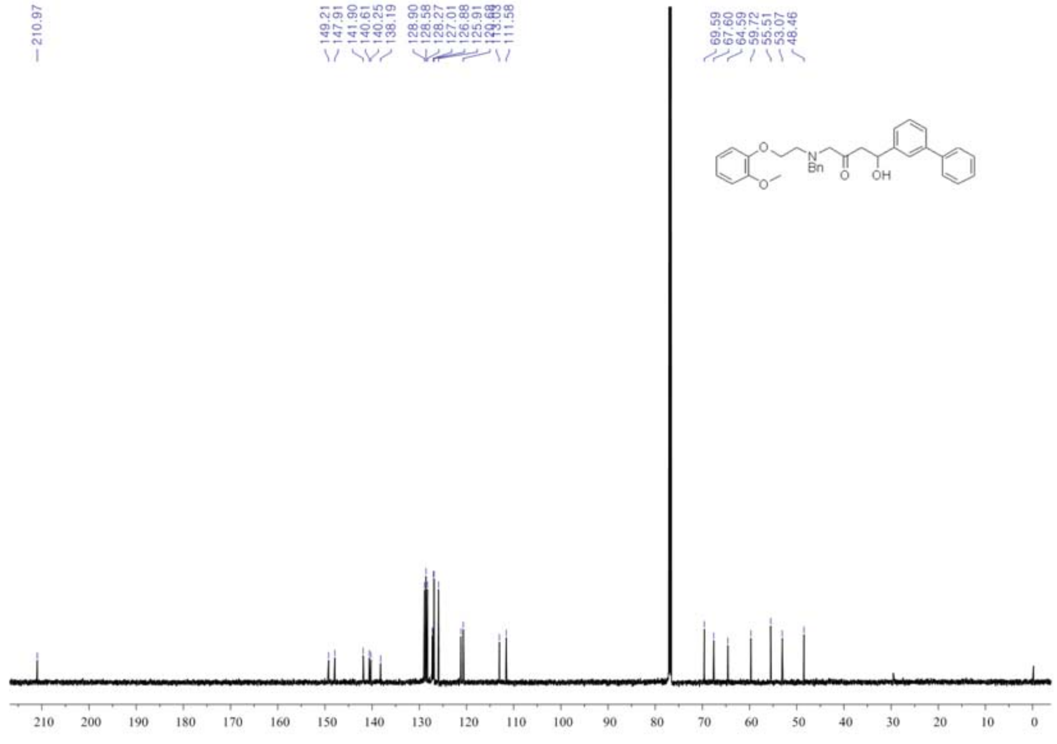

^{13}C NMR Spectrum of compound 5c in $CDCl_3$

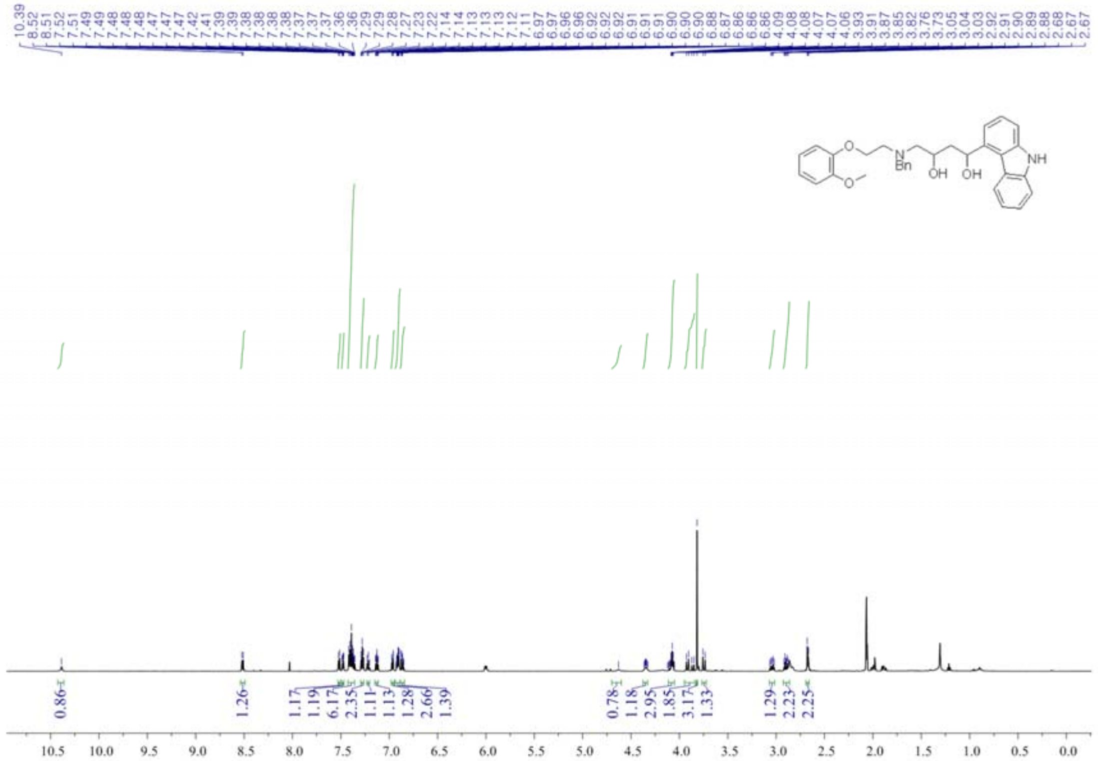

^{1}H NMR Spectrum of compound 6a in Acetoned6

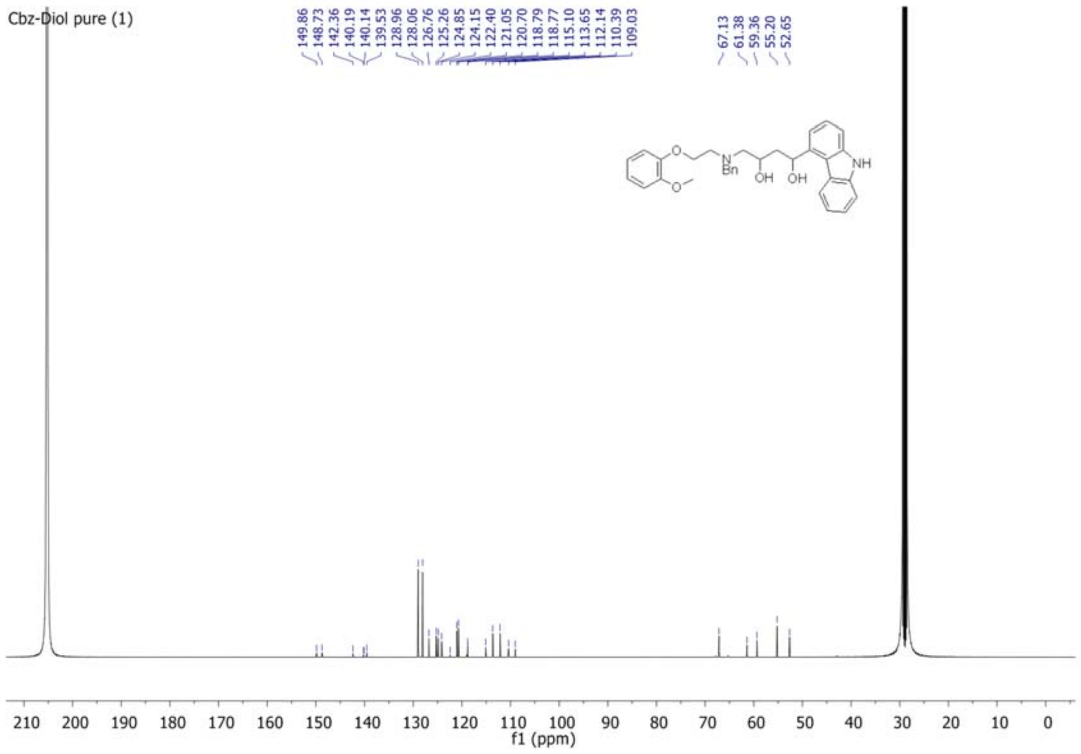

^{1}H NMR Spectrum of compound 6a in Acetoned6

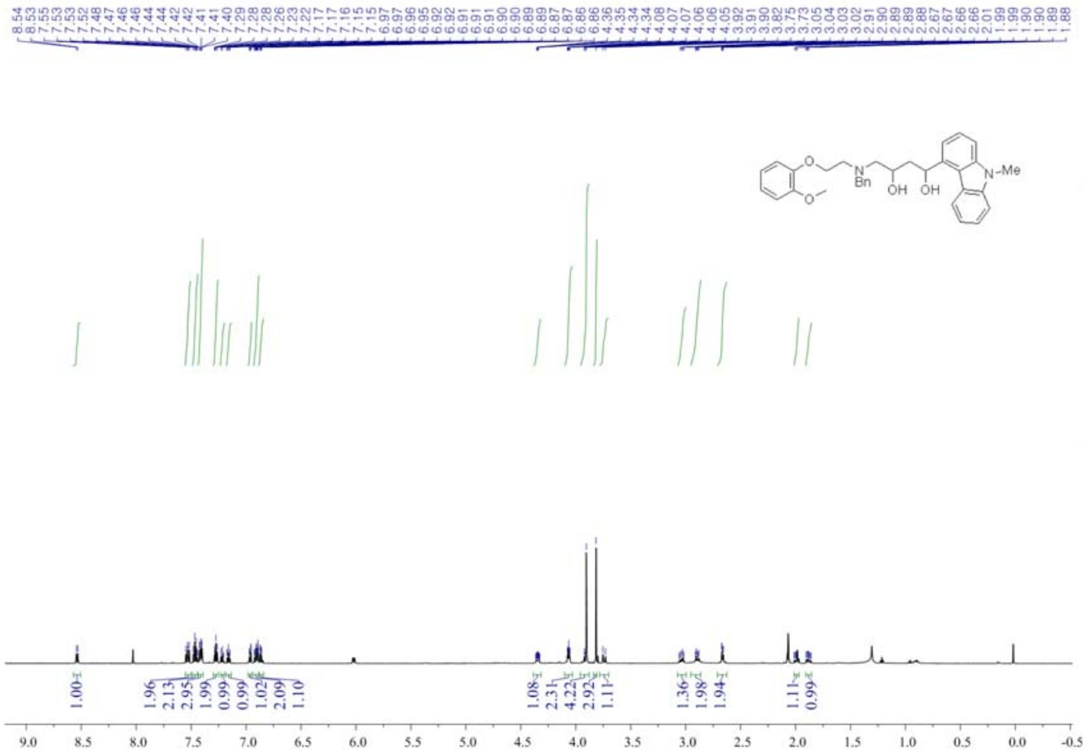

^{1}H NMR Spectrum of compound 6b in Acetoned6

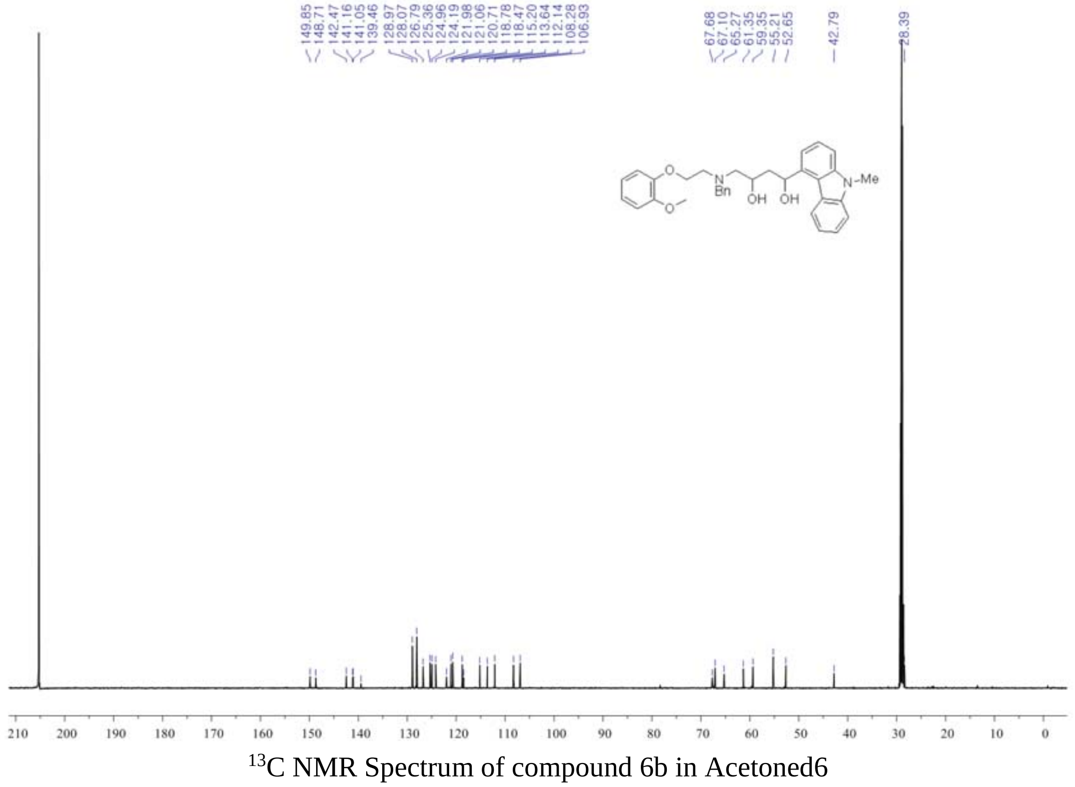

^{13}C NMR Spectrum of compound 6b in Acetoned6

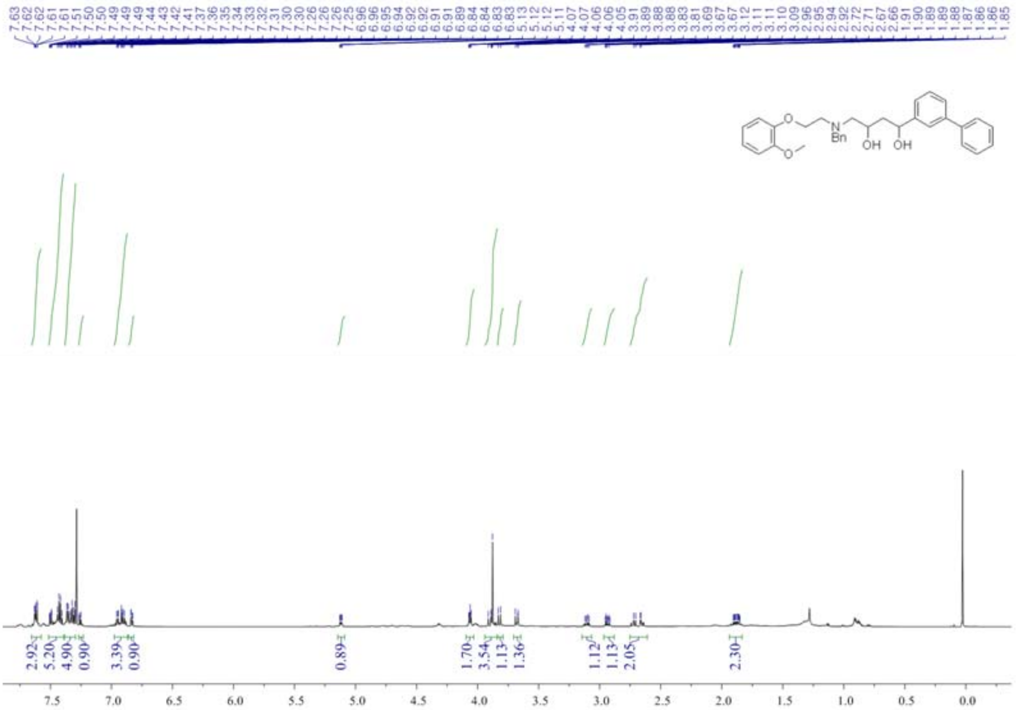

^{1}H NMR Spectrum of compound 6c in $CDCl_3$

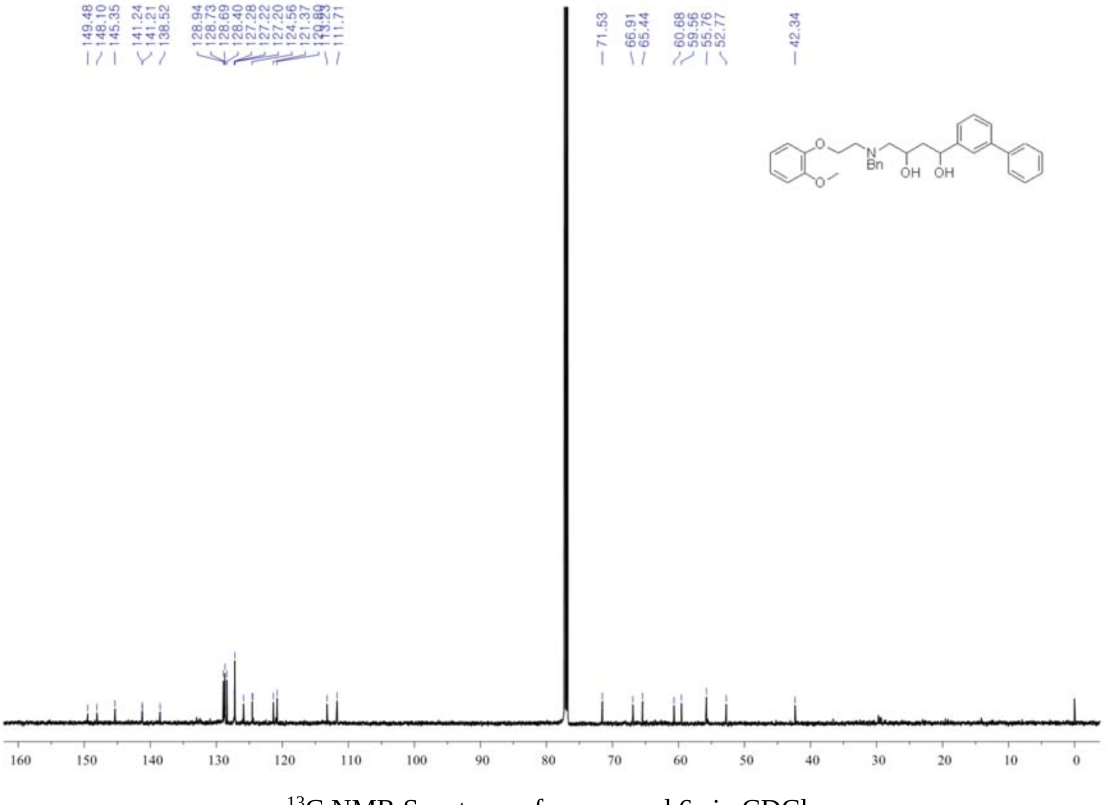

^{13}C NMR Spectrum of compound 6c in $CDCl_3$

HRMS Data

```
[ Theoretical Ion Distribution ]                                    Page: 1
Molecular Formula : C16 H19 N O2
                   (m/z   257.1416, MW   257.3324, U.S.   8.0)
Base Peak :    257.1416, Averaged MW :    257.3306(a),    257.3314(w)

    m/z        INT.
  257.1416 100.0000 ***********************************************************
  258.1448  18.2393 ***********
  259.1476   1.9647 *
  260.1502   0.1567
  261.1529   0.0098
  262.1555   0.0005
```

```
[ Elemental Composition ]
Data   : HR-NOX-1-001
Sample: -                                Date : 19-Jun-2018 14:36
Note : -
Inlet : Reserv.
RT : 1.24 min                            Ion Mode : EI+
Elements : C 17/0, H 20/0, N 1/0, O 2/0  Scan#: 38+40
Mass Tolerance        : 1000ppm, 3mmu if m/z < 3, 10mmu if m/z > 10
Unsaturation (U.S.) : -0.5 - 50.0

Observed m/z  Int%    Err[ppm / mmu]     U.S.  Composition
  257.1414   100.0      -0.7 /  -0.2      8.0  C 16 H 19 N O 2
```

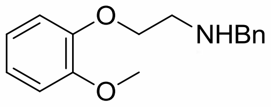

O
NHBn
O

Compound **2**

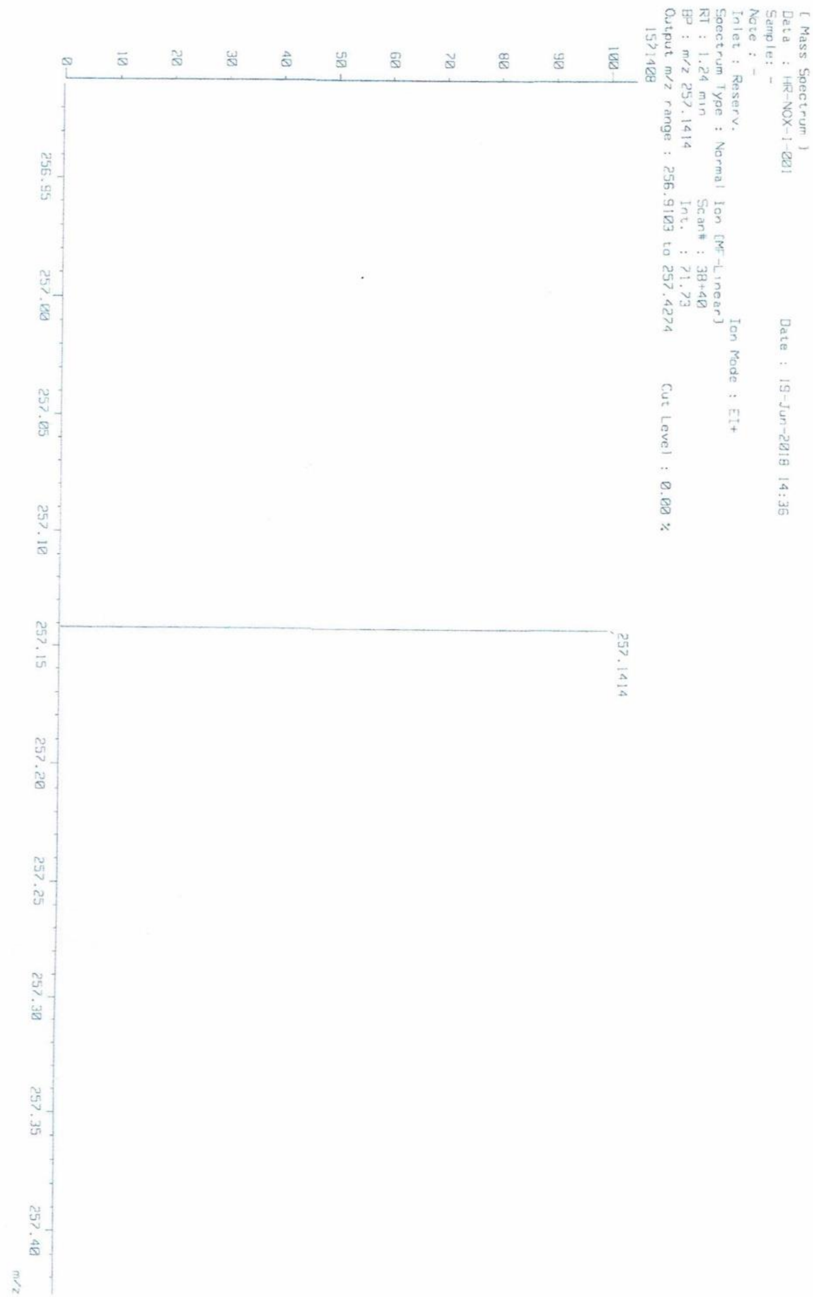

[Mass Spectrum]
Data : HR-NOX-1-001
Date : 19-Jun-2018 14:36
Sample: -
Note : -
Inlet : Reserv.
Ion Mode : EI+
Spectrum Type : Normal Ion [MF-Linear]
RT : 1.24 min
Scan# : 38+40
BP : m/z 257.1414
Int. : 71.73
Output m/z range : 256.9103 to 257.4274
Cut Level : 0.00 %
1571408
100
90
80
70
60
50
40
30
20
10
0
257.1414
256.95
257.00
257.05
257.10
257.15
257.20
257.25
257.30
257.35
257.40
m/z

[Theoretical Ion Distribution]

Molecular Formula : C19 H23 N O3
(m/z 313.1678, MW 313.3965, U.S. 9.0)
Base Peak : 313.1678, Averaged MW : 313.3943(a), 313.3951(w)

m/z	INT.	
313.1678	100.0000	**
314.1710	21.6141	*************
315.1738	2.8191	**
316.1765	0.2734	
317.1791	0.0211	
318.1817	0.0013	

[Elemental Composition]
Data : 20161229-9-4-004
Sample: -
Date : 29-Dec-2016 10:44
Note : -
Inlet : Reserv.
RT : 1.14 min
Ion Mode : EI+
Elements : C 20/0, H 25/0, N 2/0, O 3/0
Scan#: 35
Mass Tolerance : 10mmu
Unsaturation (U.S.) : -0.5 - 50.0

Observed m/z	Int%	Err[ppm / mmu]	U.S.	Composition
313.1680	26.6	+0.6 / +0.2	9.0	C 19 H 23 N O 3

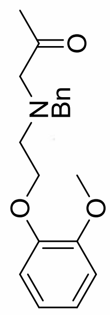

Compound **3**

[Mass Spectrum]
Data : 20161229-9-4-004 Date : 29-Dec-2016 10:44
Sample: -
Note : -
Inlet : Reserv. Ion Mode : EI+
Spectrum Type : Normal Ion [MF-Linear]
RT : 1.14 min Scan# : 35
BP : m/z 270.1558 Int. : 399.96
Output m/z range : 312.8082 to 313.5493 Cut Level : 0.00 %

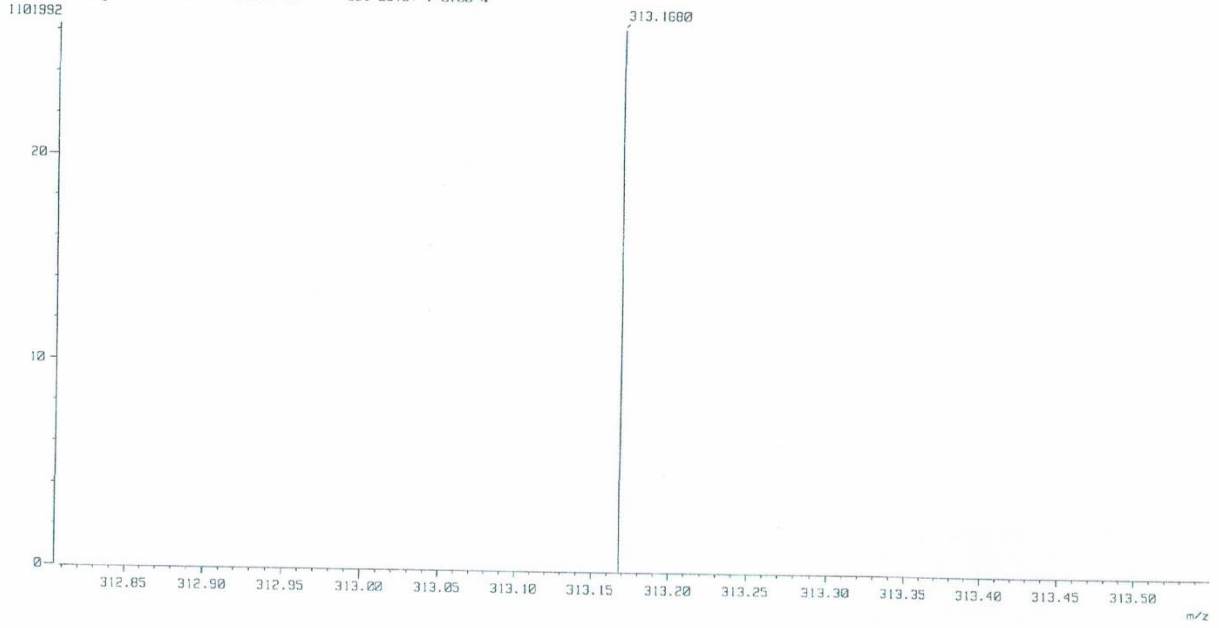

[Theoretical Ion Distribution]

Molecular Formula : C32 H32 N2 O4
(m/z 508.2362, MW 508.6171, U.S. 18.0)
Base Peak : 508.2362, Averaged MW : 508.6142(a), 508.6150(w)

m/z	INT.	
508.2362	100.0000	**
509.2394	36.4786	**********************
510.2424	7.2560	****
511.2453	1.0300	*
512.2480	0.1153	
513.2507	0.0107	
514.2534	0.0009	

[Elemental Composition]
Data : 20161229-9-5c-a-009
Date : 29-Dec-2016 11:15
Sample: -
Note : -
Inlet : Reserv.
Ion Mode : EI+
RT : 2.14 min
Scan#: 65+64
Elements : C 33/0, H 37/0, N 2/0, O 5/0
Mass Tolerance : 10mmu
Unsaturation (U.S.) : -0.5 - 50.0

Observed m/z	Int%	Err[ppm / mmu]	U.S.	Composition
508.2364	13.9	+0.3 / +0.2	18.0	C 32 H 32 N 2 O 4

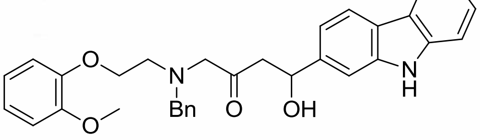
Bn
O
OH
NH

Compound **5a**

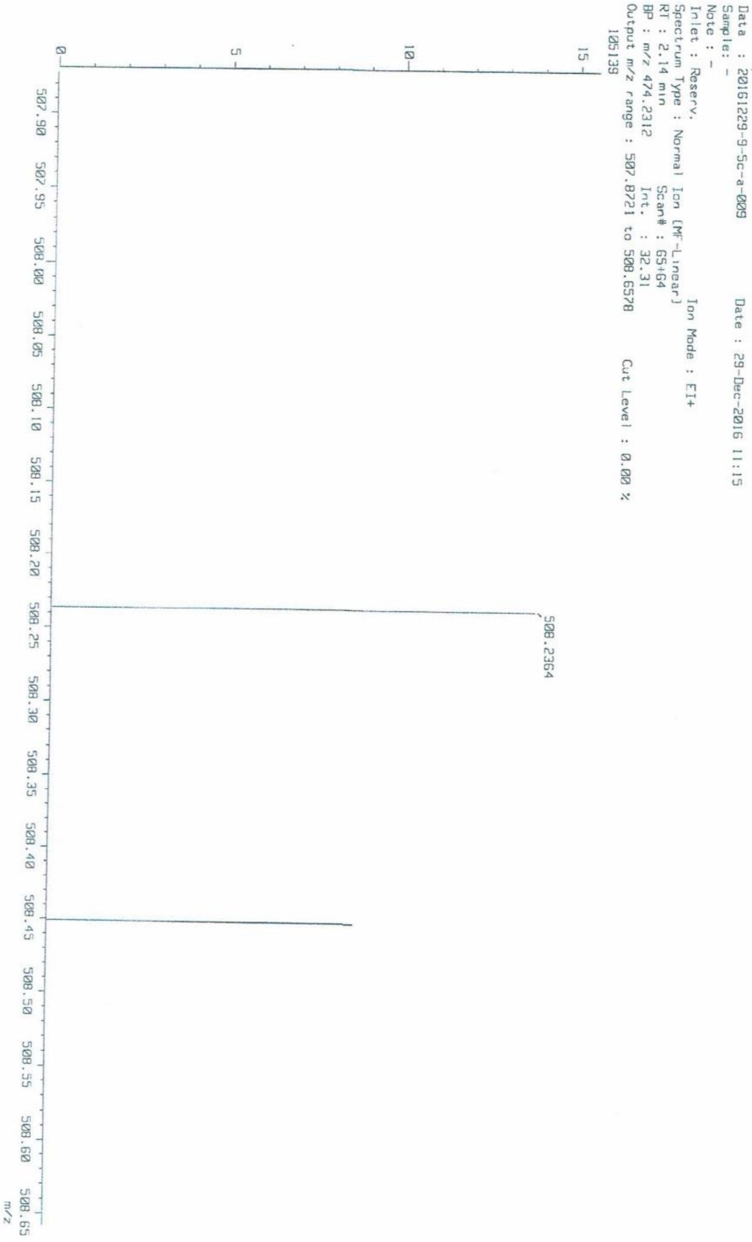
[Mass Spectrum]
Data : 20161229-9-5c-a-009
Date : 29-Dec-2016 11:15
Sample: -
Note : -
Inlet : Reserv.
Ion Mode : EI+
Spectrum Type : Normal Ion [MF-Linear]
RT : 2.14 min
Scan# : 65+64
BP : m/z 474.2312
Int. : 32.31
Output m/z range : 507.8721 to 508.6578
Cut Level : 0.00 %
105139
508.2364
m/z

[Theoretical Ion Distribution]


```
Molecular Formula : C33 H34 N2 O4
                  (m/z   522.2519, MW   522.6440, U.S. 18.0)
Base Peak :   522.2519, Averaged MW :   522.6409(a),   522.6417(w)

    m/z        INT.
  522.2519 100.0000 **********************************************************
  523.2551  37.5908 **********************
  524.2581   7.6618 ****
  525.2609   1.1107 *
  526.2637   0.1268
  527.2665   0.0120
  528.2692   0.0010
```

[Elemental Composition]

```
Data   : HR-NOX-2-002
Sample: -                                  Date : 19-Jun-2018 14:40
Note : -
Inlet : Reserv.
RT : 4.30 min                              Ion Mode : EI+
Elements : C 35/0, H 35/0, N 2/0, O 4/0    Scan#: 130
Mass Tolerance        : 1000ppm, 3mmu if m/z < 3, 10mmu if m/z > 10
Unsaturation (U.S.) : -0.5 - 50.0

Observed m/z  Int%    Err[ppm / mmu]    U.S.  Composition
  522.2518    70.5       -0.1 /  +0.0   18.0  C 33 H 34 N 2 O 4
```

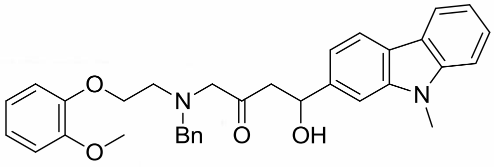

O
N
Bn
O
OH
O
N

Compound **5b**

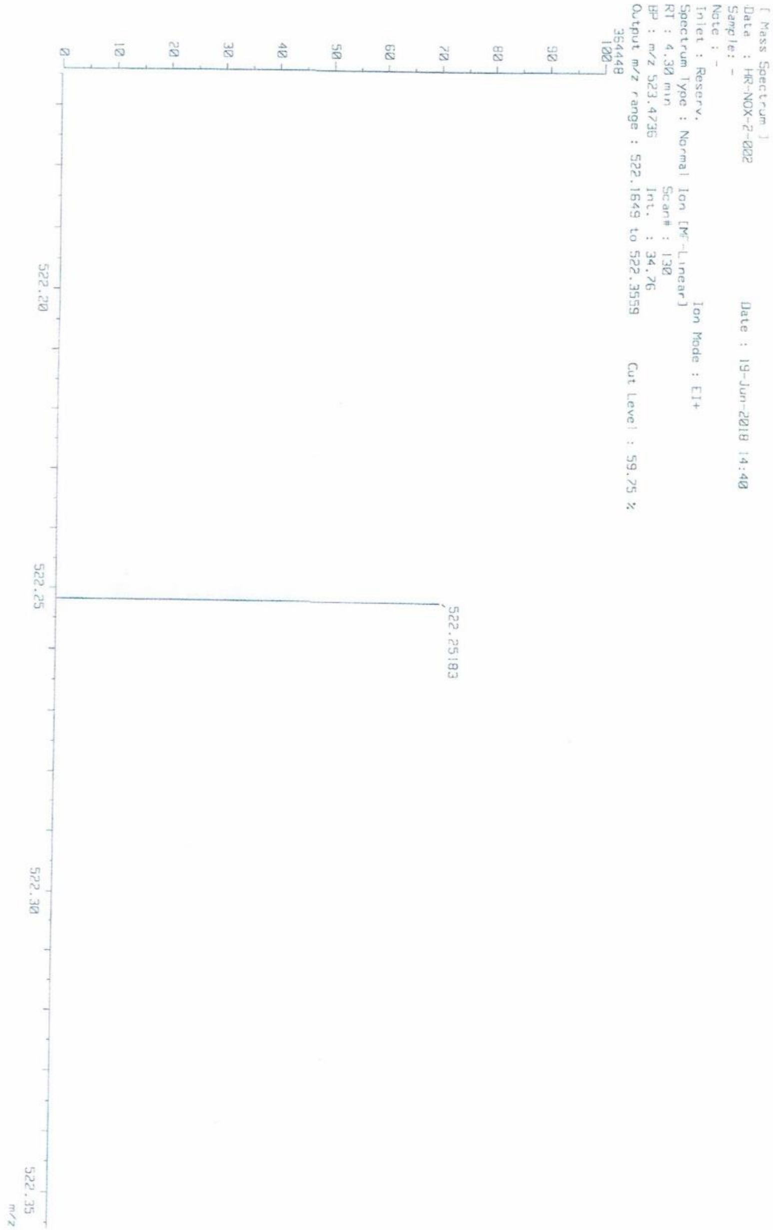

[Mass Spectrum]
Date : HR-NOX-2-002
Date : 19-Jun-2018 14:40
Sample: -
Note : -
Inlet : Reserv.
Ion Mode : EI+
Spectrum Type : Normal Ion [MF-Linear]
RT : 4.30 min
Scan# : 130
BP : m/z 523.4736
Int. : 34.76
Output m/z range : 522.1649 to 522.3559
Cut Level : 59.75 %
364448
100
90
80
70
60
50
40
30
20
10
0
522.25183
522.20
522.25
522.30
522.35
m/z

[Theoretical Ion Distribution]
Molecular Formula : C32 H33 N O4
(m/z 495.2410, MW 495.6183, U.S. 17.0)
Base Peak : 495.2410, Averaged MW : 495.6153(a), 495.6161(w)

m/z	INT.	
495.2410	100.0000	**
496.2443	36.1112	*********************
497.2473	7.1234	****
498.2502	1.0038	*
499.2530	0.1116	
500.2557	0.0103	
501.2584	0.0008	

[Elemental Composition]
Data : HR-NOX-3-006
Date : 19-Jun-2018 15:06
Sample: -
Note : -
Inlet : Reserv.
Ion Mode : EI+
RT : 2.04 min
Scan#: 62
Elements : C 33/0, H 40/0, N 1/0, O 5/0
Mass Tolerance : 1000ppm, 3mmu if m/z < 3, 10mmu if m/z > 10
Unsaturation (U.S.) : -0.5 - 50.0

Observed m/z	Int%	Err[ppm / mmu]	U.S.	Composition
495.2410	24.4	+0.1 / +0.0	17.0	C 32 H 33 N O 4

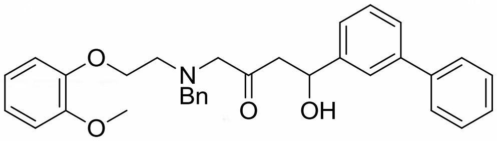

Compound **5c**

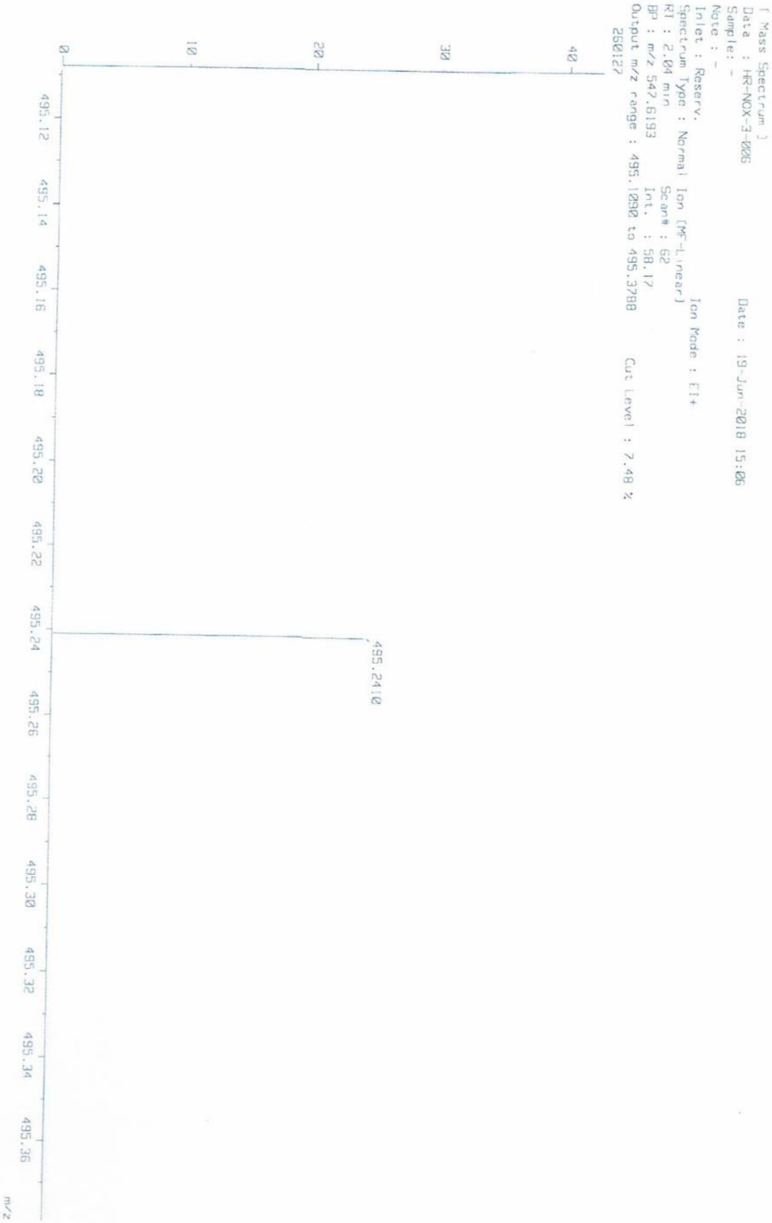
[Mass Spectrum]
Data : HR-NOX-3-006
Date : 19-Jun-2018 15:06
Sample: -
Note : -
Inlet : Reserv.
Ion Mode : EI+
Spectrum Type : Normal Ion [MF-Linear]
RT : 2.04 min
Scan# : 62
BP : m/z 547.6193
Int. : 58.17
Output m/z range : 495.1090 to 495.3788
Cut Level : 7.48 %
260127
495.2410
m/z

[Theoretical Ion Distribution]

Molecular Formula : C32 H34 N2 O4
(m/z 510.2519, MW 510.6330, U.S. 17.0)
Base Peak : 510.2519, Averaged MW : 510.6299(a), 510.6307(w)

m/z	INT.	
510.2519	100.0000	**
511.2551	36.4786	**********************
512.2581	7.2560	****
513.2609	1.0300	*
514.2637	0.1153	
515.2664	0.0107	
516.2691	0.0009	

[Elemental Composition]
Data : 20161229-9-6c-007
Sample: -
Note : -
Inlet : Reserv.
RT : 1.72 min
Elements : C 33/0, H 35/0, N 2/0, O 5/0
Mass Tolerance : 10mmu
Unsaturation (U.S.) : -0.5 - 50.0

Date : 29-Dec-2016 11:00
Ion Mode : EI+
Scan#: (52,53)

Observed m/z	Int%	Err[ppm / mmu]	U.S.	Composition
510.2522	100.0	+0.7 / +0.3	17.0	C 32 H 34 N 2 O 4

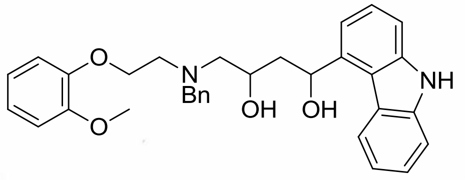
O
N
Bn
OH
OH
NH
O

Compound **6a**

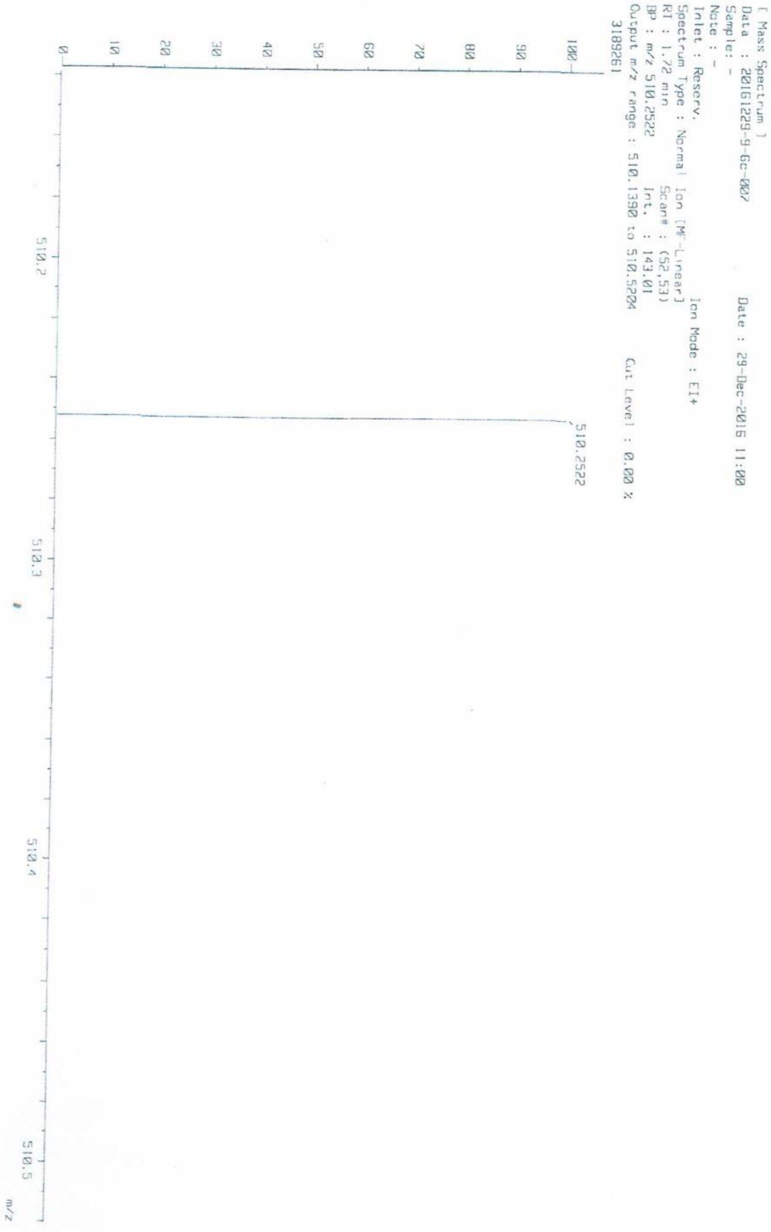
[Mass Spectrum]
Data : 20161229-9-6c-007
Date : 29-Dec-2016 11:00
Sample: -
Note : -
Inlet : Reserv.
Ion Mode : EI+
Spectrum Type : Normal Ion [MF-Linear]
RT : 1.72 min
Scan# : (52,53)
BP : m/z 510.2522
Int. : 143.01
Output m/z range : 510.1390 to 510.5204
Cut Level : 0.00 %
3189261
510.2522
0
10
20
30
40
50
60
70
80
90
100
510.2
510.3
510.4
510.5
m/z

[Theoretical Ion Distribution]

Molecular Formula : C33 H36 N2 O4

(m/z 524.2675, MW 524.6598, U.S. 17.0)

Base Peak : 524.2675, Averaged MW : 524.6566(a), 524.6574(w)

m/z	INT.	
524.2675	100.0000	***
525.2707	37.5908	***********************
526.2737	7.6618	****
527.2766	1.1107	*
528.2794	0.1268	
529.2821	0.0120	
530.2848	0.0010	

[Elemental Composition]

Data : 20161229-9-6d-008

Date : 29-Dec-2016 11:04

Sample: -

Note : -

Inlet : Reserv.

Ion Mode : EI+

RT : 5.33 min

Scan#: 161+159+161

Elements : C 33/0, H 37/0, N 2/0, O 5/0

Mass Tolerance : 10mmu

Unsaturation (U.S.) : -0.5 - 50.0

Observed m/z	Int%	Err[ppm / mmu]	U.S.	Composition
524.2677	15.5	+0.4 / +0.2	17.0	C 33 H 36 N 2 O 4

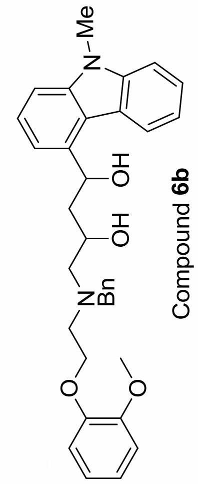

Compound **6b**

[Mass Spectrum]
Data : 20161229-9-6d-008 Date : 29-Dec-2016 11:04
Sample: -
Note : -
Inlet : Reserv. Ion Mode : EI+
Spectrum Type : Normal Ion [MF-Linear]
RT : 5.33 min Scan# : 161+159+161
BP : m/z 471.1799 Int. : 195.34
Output m/z range : 523.8819 to 524.6005 Cut Level : 0.00 %

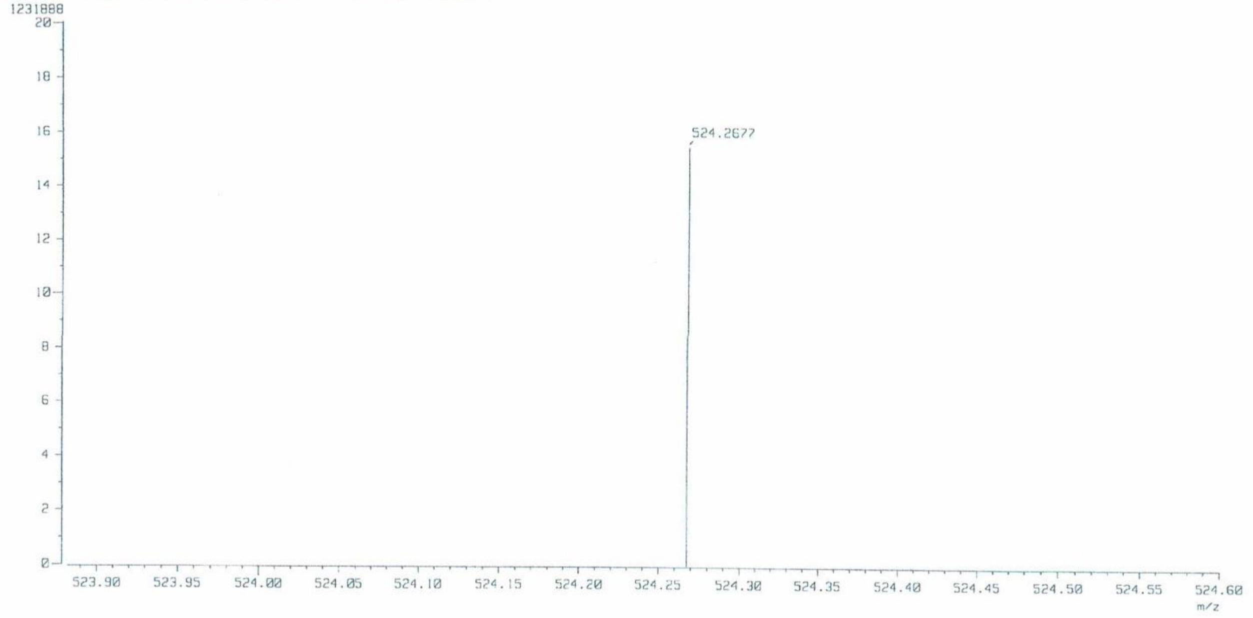

[Theoretical Ion Distribution]

Molecular Formula : C32 H35 N O4

(m/z 497.2566, MW 497.6342, U.S. 16.0)

Base Peak : 497.2566, Averaged MW : 497.6310(a), 497.6318(w)

m/z	INT.	
497.2566	100.0000	**
498.2599	36.1112	*********************
499.2629	7.1234	****
500.2658	1.0038	*
501.2686	0.1116	
502.2714	0.0103	
503.2741	0.0008	

[Elemental Composition]

Data : HR-NOX-4-005

Sample: -

Date : 19-Jun-2018 14:53

Note : -

Inlet : Reserv.

RT : 2.04 min

Ion Mode : EI+

Scan#: 62+67+69+72+75+74

Elements : C 35/0, H 35/0, N 1/0, O 4/0

Mass Tolerance : 1000ppm, 3mmu if m/z < 3, 10mmu if m/z > 10

Unsaturation (U.S.) : -0.5 - 50.0

Observed m/z	Int%	Err[ppm / mmu]	U.S.	Composition
497.2565	99.6	-0.2 / -0.1	16.0	C 32 H 35 N O 4

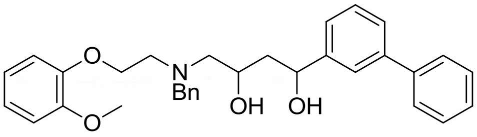

Compound **6c**

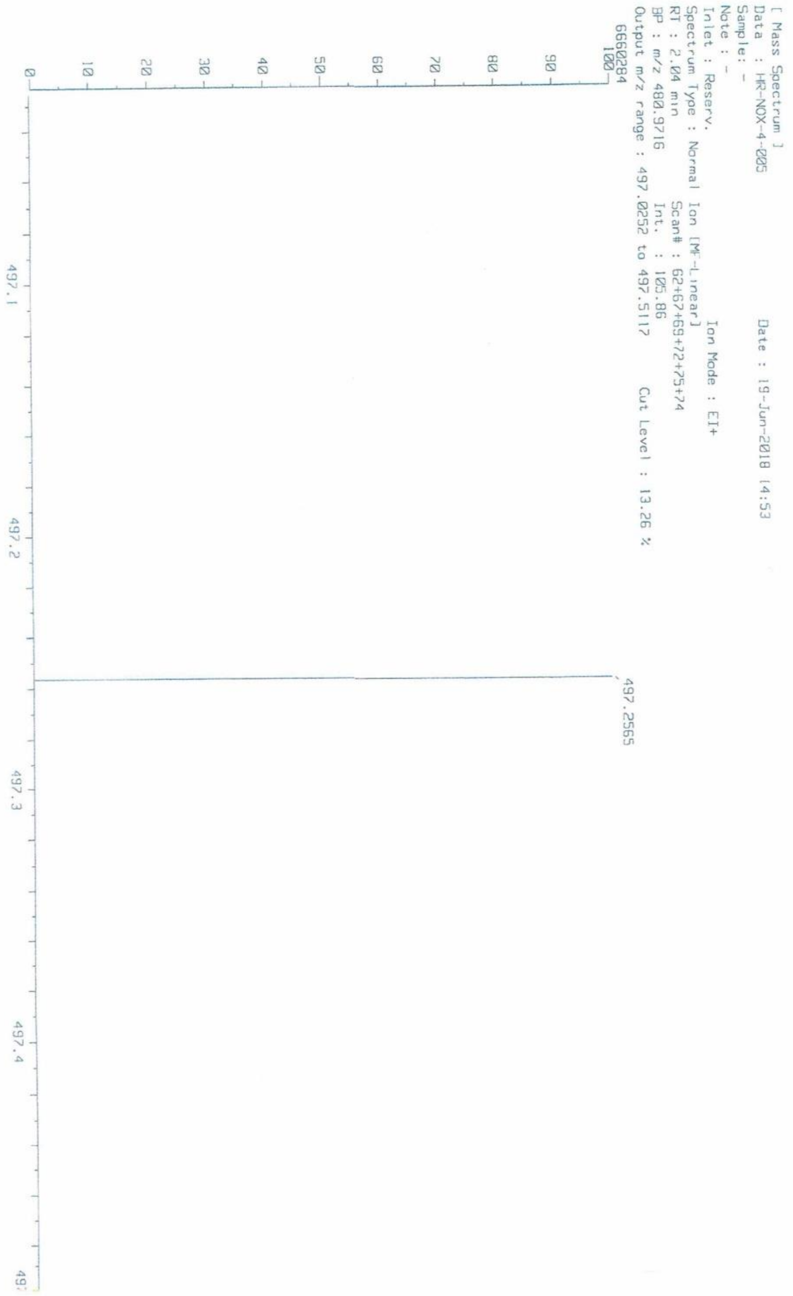
[Mass Spectrum]
Data : HR-NOX-4-005
Date : 19-Jun-2018 14:53
Sample: -
Note : -
Inlet : Reserv.
Ion Mode : EI+
Spectrum Type : Normal Ion [MF-Linear]
RT : 2.04 min
Scan# : 62+67+69+72+75+74
BP : m/z 480.9716
Int. : 105.86
Output m/z range : 497.0252 to 497.5117
Cut Level : 13.26 %
6660284
100
90
80
70
60
50
40
30
20
10
0
497.1
497.2
497.2565
497.3
497.4
